# Single base focal hypermutation cooccurs with structural variation as an early event in advanced prostate tumourigenesis with ancestry specific independence: a multi-ancestral observational study

**DOI:** 10.1186/s13073-026-01647-5

**Published:** 2026-04-25

**Authors:** Jue Jiang, Avraam Tapinos, Ruotian Huang, M. S. Riana Bornman, Phillip D. Stricker, Shingai B. A. Mutambirwa, David C. Wedge, Weerachai Jaratlerdsiri, Vanessa M. Hayes

**Affiliations:** 1 Ancestry and Health Genomics Laboratory, Charles Perkins Centre, School of Medical Sciences, Faculty of Medicine and Health, University of Sydney, Camperdown, NSW 2050 Australia; 2 Manchester Cancer Research Centre, University of Manchester, Manchester, M20 4GJ UK; 3 School of Health Systems and Public Health, University of Pretoria, Pretoria, South Africa; 4 St Vincent’s Prostate Cancer Research Centre, Sydney, NSW Australia; 5 Department of Urology, Sefako Makgatho Health Science University, George Mukhari Academic Hospital, Medunsa, Ga-Rankuwa, South Africa; 6 Computational Genomics Group, Charles Perkins Centre, School of Medical Sciences, Faculty of Medicine and Health, University of Sydney, Camperdown, NSW 2050 Australia; 7 Norwich Medical School, University of East Anglia, Norwich, UK

**Keywords:** Kataegis, Prostate cancer, Ancestral disparity, APOBEC, Cancer evolution

## Abstract

**Background:**

Kataegis, the focal hypermutation of single base positions in tumour genomes, has received little attention with regards to prostate cancer (PCa) molecular features, tumour evolution and associated clinical presentation. Most notably, the impact of this phenomenon is yet to be explored across ancestral lineages representing the extremities of PCa presentation and outcomes, with men of African ancestry disproportionately disadvantaged. The purpose of this study is to address the knowledge gap through African inclusive multi-ancestral interrogation.

**Methods:**

We assessed for ancestrally shared and unique molecular, evolutionary and clinical features of kataegis in 669 multi-ancestral whole PCa genomes. Access to raw whole-genome sequenced data allowed for direct single-pipeline comparative analysis between 109 southern African and 57 European derived treatment naïve high-risk-biased primary tumours (74% and 88%) with paired blood samples, further assessed against publicly available 207 Asian high-risk-leaning comparative (65%) and 296 European low-risk-biased alternative (79%) resources. Comparisons between ancestries and risk groups were through Wilcoxon’s rank sum test and Fisher’s exact tests, with *P* values adjusted by false discovery rate.

**Results:**

Confirming relatively low burdens, we found kataegis to be significantly associated with genomic instability, cancer drivers, and clinical adversity across ancestries (false discovery rate = $\:7.44\times\:{10}^{-6}-0.04$). Notably, kataegis-postive tumours were associated with elevated prostate-specific antigen levels at presentation in African (false discovery rate = $\:1.66\times\:{10}^{-3}$) and higher risk for metastatic progression in European patients (Kaplan-Meier estimator, $\:P=0.03$). Enrichment of APOBEC’s context preferences showed more attribution from APOBEC3B than APOBEC3A. Further through analyses of evolution and structural variant (SV) co-occurrence, commonly the ancestry agnostic SV-associated kataegis predominated in the clonal evolutionary state, while the less common the SV-independent kataegis ($\:P=0.03$) and subclonal kataegis ($\:P=1.67\times\:{10}^{-3}$) showed African specificity.

**Conclusions:**

We found kataegis-positivity to be associated with poor PCa presentation and prognosis, irrespective of patient ancestry. Kataegis-related genomic instability occurring early and late during African derived tumourigenesis, may partly explain the heightened tumour and clinical heterogeneity observed for patients of African ancestry.

**Supplementary Information:**

The online version contains supplementary material available at 10.1186/s13073-026-01647-5.

## Background

Prostate cancer (PCa) is the most frequently diagnosed male cancer in most regions of the world, disproportionately affecting men of African ancestry and particularly from Sub-Saharan Africa [1]. Mortality rates from PCa are highest in Sub-Saharan Africa and the Caribbean, with southern Africa ranking the first globally at 29.7 (age-standardised rate per 100,000 males) [1]. Notably, the incidence rate of southern Africa is lower than that of economically stable regions, such as Australia and New Zealand (59.9 vs. 78.1, age-standardised rate per 100,000) [1]. Conversely, both incidence and mortality rates are lowest across the Asian diaspora of nations. While the disparities may be attributed to diminished access to PCa screening and medical resources or exposure to yet unknown geographic risk factors, studies from the United States have shown that African American men are at greatest risk for aggressive disease presentation and associated lethality after accounting for non-genetic factors [2, 3]. Additional studies that alluded to biological and genomic contributions are needed for a better understanding of the disparities across different ancestral populations.

Kataegis, meaning thunderstorm in Greek, describes the focal hypermutation phenomenon in cancer genomes [4]. A kataegis event is defined as a cluster of closely distributed single nucleotide variants (SNVs) and results from a single mutational action of APOBEC3A (A3A) or APOBEC3B (A3B) cytidine deaminases on exposed single-strand DNAs (ssDNAs) [4–6]. This mutational process has been linked to single base substitution (SBS) signatures, SBS2 and SBS13 [7]. Despite Pan-Cancer Analysis of Whole Genomes (PCAWG) and organ-specific studies suggesting kataegis to be frequent in cancers of the breast, bladder, lung, and skin (melanoma) [4, 5, 8, 9], the evolution of kataegis and its clinical implications remain elusive for PCa, and unclear for African patients due to a lack of African-derived whole tumour genome data [10, 11]. Controversially, breast cancer (BRCA) research reported kataegis with a favourable prognosis [12] and lower genomic instability [10, 12], with others showing a link to aggressive disease [7]. The early event of kataegis arising with chromothripsis during telomere crisis has been suggested by modified cell line experiments [13, 14], while late kataegis development is observed in PCa [15] and hepatocellular carcinoma [8]. However, the potential contribution and association of kataegis in PCa ancestral disparities are yet to be determined.

This study aims to characterise kataegis mutational processes in PCa genomes from patients of different ancestries and to assess the potential clinical implication, with a particular focus on aggressive disease in African men. We processed samples from 109 African men (Black South Africans) and 57 European men (predominantly Australians) through the same pipeline, providing a direct comparative analysis based on genetic ancestry. Across ancestries, our findings linked kataegis events with more aggressive PCa manifestations and adverse clinical outcomes. The investigation of the aetiology primarily attributed kataegis to APOBEC enzymes with variation between cancer aggressiveness among African patients. We observed ancestral disparities in the evolutionary timing of kataegis and the distribution of distances between kataegis and structural variants (SVs). These findings highlight the unique genetic factors contributing to PCa in African men and underscore the importance of including diverse ancestral populations in cancer research.

## Methods

### Subjects, clinicopathology and whole genome sequenced resource

Treatment naive samples of blood and tumour pairs were collected from 166 patients diagnosed with PCa recruited from South Africa (*n* = 113) and Australia (*n* = 53, Table 1). Patients were recruited either at time of diagnostic biopsy from a participating Southern African Prostate Cancer Study (SAPCS) urology clinic (South Africa) or at time of surgery from St Vincent’s Prostate Cancer Clinic (Australia). Patient ancestry was determined through the interrogation of 7,472,833 markers for subpopulation fraction analyses using the fastSTRUCTURE logistic prior model [16], as previously described [17]. In short, 109 patients categorised as African (all South African) with greater than 85% African ancestral fraction; 57 were categorised as European (53 Australian and 4 South African), allowing up to 3% African ancestral and 26% Asian contributions. Tumour aggressiveness was defined from histopathological Gleason Scores as the International Society of Urological Pathology (ISUP) Grade Group (GG) and defined further as low-risk (LR, GG1 and GG2) or high-risk (HR, GG3–5). As the African derived HR group biased towards very HR PCa (89%, 72/81 ISUP GG4/5), we intentionally selected untreated biobanked samples with advanced disease for our European cohort (98%, 49/50 ISUP GG 4/5). As previously reported for South-East Africa [18], both prostate specific antigen (PSA) levels (median 82.60 vs. 8.15 ng/mL) and age at presentation/surgery (median 69 vs. 63 years in HR groups) are elevated for our African over the European cohort of HR groups. The latter cohort allows for extensive follow-up data defined as biochemical relapse (BCR) and/or metastasis (median ± s.d., 122.5 ± 44.4 months) [17].

**Table 1:** Demographic and clinical information of the current study

Ancestry	Cohort size	Cohort size per country (%)	Cohort size of low-risk (GG1–2, %)	Cohort size of high-risk (GG3–5, %)	Median age (range)
The study cohort					
Total	166	113 (68%) South Africa, 53 (32%) Australia	35 (21%)	131 (79%)	65 (45–99) ^a^
African	109	109 (100%) South Africa	28 (26%)	81 (74%)	68 (45–99) ^a^
European	57	4 (7%) South Africa, 53 (93%) Australia	7 (12%)	50 (88%)	63 (46–72)
Public validation cohorts					
European	296	296 (100%) Canada	234 (79%)	62 (21%)	64 (42–81)
Asian	207	207 (100%) China	73 (35%)	134 (65%)	69 (50–88) ^a^

^a^ One patient with missing age excluded

DNA extracted from fresh frozen tumour and patient-matched whole blood or buffy coat underwent deep whole genome sequencing (WGS) using the Illumina NovaSeq and Hiseq platforms (median coverages tumour 88.64 X and blood 44.19X), with data processed using an African-inclusive high throughput variant calling pipeline [17, 19]. In brief, the mapping of WGS data to the GRCh38 reference was through BWA-MEM [20]. The process was achieved in high-level parallelism using fastp for physical data chunking [21]. The refinement of BAM files utilised SAMBLASTER [22] to mask duplicate reads and GATK v4 base quality score recalibration (BQSR) to lessen the impact of systematic errors introduced during sequencing. Short variant calling followed the GATK pipelines: germline short variant discovery (SNPs + Indels) and somatic short variant discovery, with annotation using GATK Funcotator [23]. SV was called as a consensus call set from GRIDSS [24] and Manta [25] callers. The estimation of evolutionary timing pipeline used PhyloWGS [26] to construct subpopulations of SNVs based on the variant allele frequency, corrected by allele-specific copy number changes estimated by TITAN [27] and tumour purity. The estimation of evolutionary timing of subpopulations of SNVs used MutationTimeR [28], which calculates possibilities of epochs per SNV from a hierarchical model assuming a beta-binomial distribution.

### Public validation cohorts

Somatic SNVs were downloaded from published deep WGS primary tumour-normal data derived from 296 European and 207 Asian PCa donors, with available clinical data (Table 1). European data were derived from the Prostate Adenocarcinoma Canada project via the International Cancer Genome Consortium (ICGC) Data Portal [29, 30]. Asian data were obtained from the Chinese Prostate Cancer Genome and Epigenome Atlas (CPGEA) with accession number PRJCA001124 [31]. The European data are biased towards the LR PCa, with no age differences between LR and HR cases for either European data (79%, *n* = 234 vs. 21%, *n* = 62; median of age, 64 vs. 63.5 years; Wilcoxon’s rank sum test, *P* = 0.58) or Asian data (35%, *n* = 73 vs. 65%, *n* = 134; the same median of age at 69 years).

### Kataegis identification and evolution

Kataegis identification followed the methods of the PCAWG study, using an adjusted threshold for candidate calling, followed by two criteria (detailed in Additional file1: Supplementary methods) [5]. Briefly, inter-mutational distances of SNVs were adjusted with the piecewise constant fitting (PCF) model using the core algorithms of the kataegis package [32] with default parameters [9]. The threshold, requiring a minimum of four SNVs with the PCF-adjusted distances less than one kb, was set and derived from the total number of SNVs per patient and identical for all patients.

Kataegis events were further refined with evolutionary timing (detailed in Additional file1: Supplementary methods). As kataegis SNVs arise together from a single mutational process [5], we refined kataegis with evolution by examining each subset of SNVs that occurred during the same evolutionary epochs, including clonal (early, late, and unspecified) and subclonal epochs. Clonal SNVs were regarded as originating from the clonal tumour cells. Among them, unspecified clonal SNVs were in diploid chromosomal regions. In regions where copy number was gained, early and late clonal SNVs were discerned to show origins before and after the copy number gain, respectively. This step was applied only to the current study cohort and identified a total of 249 evolutionary kataegis events in 65 patients. Evolutionary kataegis was unavailable for public cohorts due to the lack of available copy number variants (CNVs).

### Statistical analysis

Statistical tests included Fisher’s exact test for categorical variables using the stats package [33], and Wilcoxon’s rank sum test for continuous data comparisons between two ancestries or risk groups using the ggpubr package (v0.6.0) [34] in R (v 4.2.2) [33]. *P*s of multiple hypothesis testing were adjusted using the false discovery rate (FDR) when specified. Four outliers with extreme kataegis burdens were excluded, including one European patient (42 kataegis events) in the study cohort, and three patients whose z-scores were greater than three in the public European cohort.

For genomic features significantly associated with the presence of kataegis, we further analysed their associations with kataegis burden with a negative binomial regression model. The negative binomial regression model was suitable to describe the kataegis burden that had many zero values and a variance greater than its mean (4.03 vs. 1.03). The analysis excluded the aforementioned outlier, and three African patients with PSA or age unavailable. Besides all the genomic features associated with kataegis, the analysis also included ancestry, patient risk levels and age at diagnosis. Log-transformation was applied to adjust data skewness found in SV burden, tumour mutational burden (TMB), chromothripsis burden, percentage of genome alteration (PGA), and copy number (CN) gain.

### Co-occurrence of kataegis with cancer driver mutations

We examined associations between kataegis and point mutations of 58 selected genes using Fisher’s exact test. We examined previously reported top cancer drivers for PCa [17, 35] and/or genes potentially related to kataegis development, such as cell-cycle checkpoint-related genes [36], *APOBEC3A*, and *APOBEC3B*.

### Survival analysis

We performed survival analyses using Kaplan-Meier estimates from the survival package (v 3.5-5) [37] and log-rank tests from the survminer package (v 0.4.9) [38]. To assess clinical progression, we compared (i) patients with BCR and/or metastasis to those with neither, and (ii) patients with metastasis only to those without metastasis or BCR. The survival distribution was compared by kataegis state (positive or negative), and by kataegis burden (elevated burden with a kataegis count > 1 or ≤1). The analysis was performed for LR and HR groups concurrently and separately for the European patients with available follow-up data from our study cohort, and for public European and Asian cohorts. From our study cohort, we excluded the small LR group of European patients (*n* = 7), a hyper-kataegic outlier, and three patients not curative after radical prostatectomy from the HR group (*n* = 42 remaining). From the validation cohorts, we excluded three outliers defined by z-scores greater than three, and patients with missing clinical follow-up from the public European cohort (*n* = 281 remaining). We also filtered out 21 patients with missing clinical information from the Asian cohort (*n* = 186 remaining).

### SBS and SV signatures

Kataegic SNVs, genome-wide SBS, and SV signatures were decomposed and assigned using SigProfilerExtractor (v.1.1.24) [39]. The analysis processed kataegic SNVs from 283 kataegis positive tumours from this study and validation cohorts. The aforementioned outliers, one from the study cohort and three from the public European cohort, were excluded from the analysis. Kataegic SNVs from the public European data were lifted to GRCh38 reference using liftOver (last modified 2022-01-31) [40]. The signature identification steps included de novo signature discovery using nonnegative matrix factorisation (NMF) and the assignment of conventional Catalogue Of Somatic Mutations In Cancer (COSMIC) signatures (v3.4, Oct. 2023). We used default settings with some modifications, including a maximum of 15 signatures, 500 NMF replicates, one million maximal NMF iterations, and the GRCh38 reference. The assignment of SBS signatures was challenging for kataegic SNVs due to a small number of SNVs compared to genome-wide SNVs. To maintain the accuracy, 33 samples were filtered out from a cut-off of cosine similarity greater than 0.5. The passed samples had a median cosine similarity of 0.851 (range, 0.508–0.988). In addition, genome-wide SBS and SV signatures were identified from 165 samples, excluding a European outlier. The SigProfilerExtractor parameters and version of the COSMIC database were the same as those used for the kataegic SBS signatures. The NMF extraction methods were based on the frequency matrix of 32 SV types [41].

### APOBEC attribution to kataegis

We used Fisher’s exact test to identify APOBEC-enriched kataegis, which were further tested for A3A or A3B enrichment according to the context preference of APOBEC enzymes. The identification mainly followed the method previously used for genome-wide enrichment [42]. For the APOBEC enrichment, kataegis events were compared with other non-clustering SNVs from the sample for the count of mutated cytosines in each motif (C and TCW) adjusted by the accessible rate of the motif ($\:\pm\:$20 bp context). Here, we used TCW to represent the APOBEC enzyme preference motif, as observing comparable amounts of cytosine mutations in TCA and TCT, rather than a skewness toward TCA reported previously [42]. We used TCW to represent a cytosine mutation in the TCW motif, and more details of the Fisher’s exact test are in Additional file1: Supplementary methods. Further, for each APOBEC-enriched kataegis, we identified A3A-enriched kataegis with YTCW motif and A3B-enriched kataegis with RTCW motif [42, 43], where the underlined cytosine means mutated. *P*-values were adjusted with FDR.

### Distribution of kataegis and proximal SVs

The enrichment or sparsity of SVs proximal to kataegis events was tested by comparing kataegis with simulated kataegis events. For each kataegis event (*n* = 831) identified in this study and validation cohorts, excluding four outliers, we simulated 1,000 pseudo kataegis events with the same event interval by randomly assigning the central position with 1,000 non-clustering SNVs from the sample. For both identified kataegis and simulated kataegis, their distances to proximal SVs were compared using log-spaced bins (0–1 kb, 1 kb – 10 kb, 10 kb – 0.1 Mb, 0.1 Mb – 1 Mb, 1 Mb – 10 Mb, 10 Mb – 100 Mb, and beyond 100 Mb). For each patient group defined by ancestry and risk level, we tested enrichment or sparsity of SVs by calculating *P*-values based on the rank of the identified kataegis in the 1,000 simulated kataegis events. *P*-values were adjusted with FDR.

## Results

### Ancestrally independent low prevalence and burden for prostate tumour kataegis

**Fig. 1:**
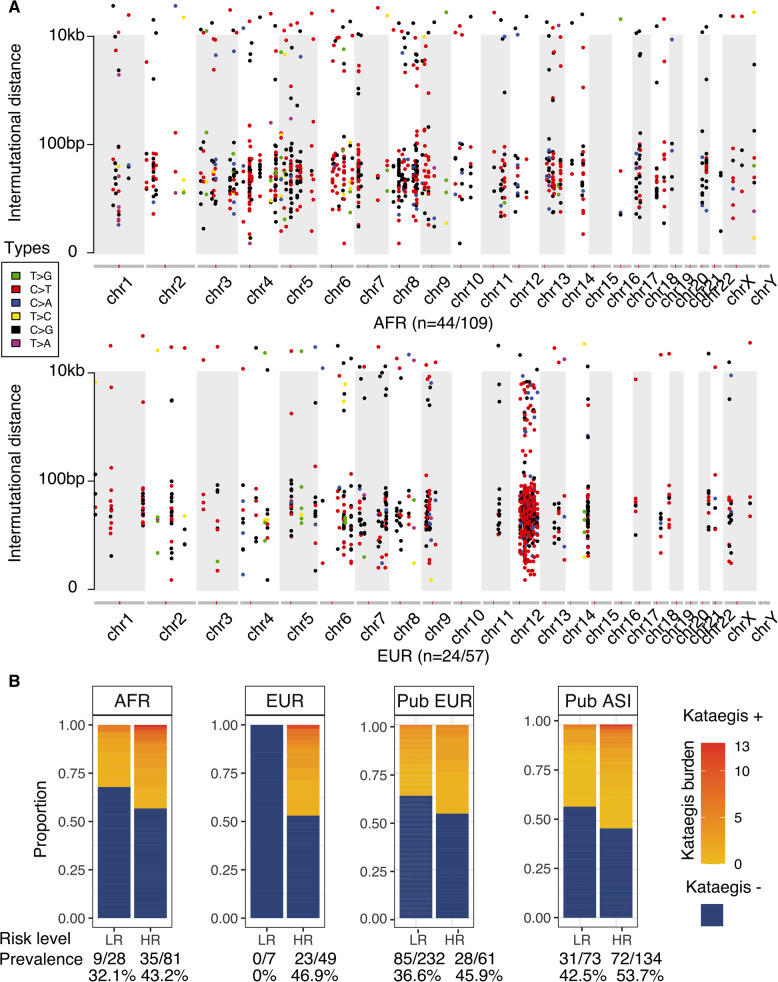
Distribution and prevalence of kataegis in prostate tumours.** A** Distributions of clustered kataegic SNVs identified among 44 African and 24 European patients from this study cohort (*n* = 166). The extensive kataegis burden of chromosome 12 in European patients is driven by a single outlier. **B** Prevalence of kataegis and kataegis burden by patient ancestry. The presence of kataegis is defined as negative (kataegis -, dark blue) and positive (kataegis +), which are further indicated by a low to high kataegis burden (yellow to red gradient), with hyper-kataegis outliers excluded from the analysis. Patient ancestries are labelled as African (AFR), European (EUR), and Asian (ASI), with the prefix ‘Pub’ added for public data. Cancer risk levels are defined as low-risk (LR, ISUP GG1–2) and high-risk (HR, ISUP GG3–5) clinicopathological presentation. Numbers underneath define the number of kataegis + tumours vs. the total number of tumours and the prevalence, with hyper-kataegis outliers excluded

From the study cohort including 113 Africans from South Africa, 53 Europeans from Australia, and validation cohorts 296 Europeans from Canadian, 207 Asian from China (Table 1), we identified kataegis with TMB-derived threshold and criteria based on known kataegis characteristics [5]. For the study cohort, 260 kataegis events were identified in 41% (68/166) of tumours (Fig. 1A, Additional file2: Table S1), similar frequency as previous studies (Additional file2: Table S2) [5, 7, 8, 44]. Within the validation cohorts, we identified 321 kataegis events in 39.2% (116/296) of European and 297 events in 49.8% (103/207) of Asian patients (Additional file2: Table S3, S4). Overall, we observed a low kataegis burden (median: two events, range: 1 to 13, Fig. 1B), excluding one hyper-kataegic outlier (47 events) derived from a single European patient. The median number of SNVs of a kataegis event is six, spanning a narrow range of 2.67 kb and differing between HR groups by ancestry (African 5 SNVs vs. European 7 SNVs, Wilcoxon’s rank-sum test, FDR = $\:4.49\times\:{10}^{-4}$). Kataegic regions were unique to each patient, as previously described [45], and only a few were within functional genomic regions (Additional file2: Table S5).

### Kataegis is associated with genomic instability and co-occurs with cancer drivers

Kataegis-positive tumours exhibited increased genomic instability marked by various genomic features observed in one or more groups of risk levels and genetic ancestries with Wilcoxon rank sum test (Additional file1: Fig. S1). Elevated TMB, SVs, and chromothripsis were observed in kataegis-positives across ancestries and cancer aggressiveness (FDRs = $\:2.53\times\:{10}^{-5}$–0.02, $\:7.44\times\:{10}^{-6}$–$\:2.20\times\:{10}^{-3}$, and $\:2.05\times\:{10}^{-5}$–$\:2.33\times\:{10}^{-3}$, respectively). Notably, the SV burden was the most significant factor, showing a further association with kataegis burden in both ancestral groups (negative binomial model, FDR = 0.01), consistent with the previous study of European patients [5]. CNVs were significantly correlated with kataegis exclusive to HR groups of African and European patients, characterised by gains (FDRs = 0.01, 0.04), losses (FDRs = $\:1.67\times\:{10}^{-4}$, $\:1.66\times\:{10}^{-3}$) or both as measured by PGA (FDRs = $\:2.47\times\:{10}^{-4}$, $\:1.66\times\:{10}^{-3}$). Significantly shorter telomere lengths were observed in kataegis-positive tumours derived from African patients within the LR group (FDR = 0.03).

Significant co-occurrences of kataegis and cancer driver point mutations were observed using Fisher’s exact test (Additional file2: Table S6). The significant co-occurrence of kataegis and *RBFOX1* in HR groups was found for both ancestries (FDRs = $\:7.34\times\:{10}^{-4}$, $\:2.71\times\:{10}^{-3}$) and validated by the LR group of public European patients (*n* = 234, FDR = $\:2.98\times\:{10}^{-6}$). Additionally, significant on-occurrence of kataegis with *PDE4D*,* TP53*, and *ZFHX3* were observed in the HR group of European patients of the study cohort (FDRs = $\:4.77\times\:{10}^{-4}$, 0.04, $\:4.72\times\:{10}^{-3}$, respectively), as well as *ATM*, *ATRX*, and *CHEK2* observed the LR group of the public European patients (FDRs = $\:1.73\times\:{10}^{-3}$, 0.03, and 0.03, respectively). However, no significant co-occurrence was found in the public Asian cohort (*n* = 207, FDR > 0.3).

### Kataegis correlates with adverse PCa clinical outcomes

**Fig. 2:**
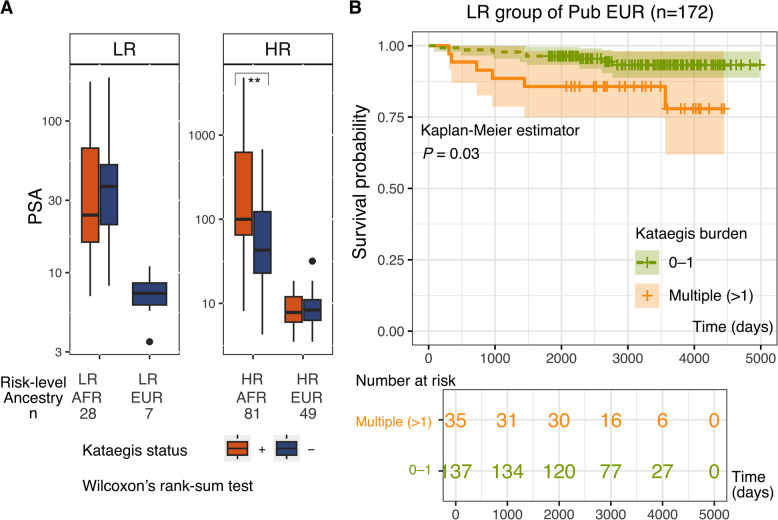
Kataegis implication in clinical measurements and outcomes.** A** Prostate-specific antigen (PSA) present in 165 PCa patients and distinguished by clinical risk and genetic ancestry. A hyper-kataegis outlier is excluded. PSA values are compared between kataegis positive (+) and negative (-) within particular risk levels, defined as low-risk (LR, ISUP GG1–2) and high-risk (HR, ISUP GG3–5) clinicopathological presentation, and by patient ancestry (AFR, African and EUR, European). The number of patients per group is labelled underneath, excluding an outlier of extremely high burden of kataegis. Significant result of Wilcoxon’s rank sum test is defined by false discovery rate (FDR); ns, not significant; and **, FDR = $\:1.66\times\:{10}^{-3}$. **B** Kaplan-Meier survival estimates correlating kataegis abundance within the low-risk (LR) group of public European data with clinical follow-up (time in days, *n* = 172). The comparisons are between patients having multiple kataegis (kataegis = multiple, *n* = 35) against having no or one kataegis (kataegis = 0–1, *n* = 137). Clinical outcomes analysed are defined as metastasis. Two outliers with z-scores greater than three and one with missing metastasis data were excluded (see Materials and methods). Patients with biochemical relapse and no metastasis were excluded

To study the clinical implication of kataegis, we examined the PSA level of patients, a widely used clinical measurement for PCa detection [46] and post-treatment recurrence [47]. Higher PSA levels were observed with kataegis positive tumours compared to those with negative tumours in the HR group of African patients (median: 100 vs. 43.0 ng/mL; Wilcoxon’s rank-sum test, FDR = $\:1.66\times\:{10}^{-3}$; Fig. 2A). Appreciating that high PSA levels may be an indicator of metastasis risk [46], lack of associated clinical follow-up data, including associated magnetic resonance imaging (MRI) data, limited further investigation. For the HR group of our European patients sampled at surgery, neither prevalence nor burden of kataegis was a significant predictor of BCR (Kaplan-Meier test), likely due to their small cohort size. Leveraging a larger LR-biased European PCa data resource, we showed LR patients with elevated kataegis burden (more than one event) to be significantly susceptible to metastasis (Log-rank test, *P* = 0.03), while observing no association for BCR (Fig. 2B).

### APOBEC3B is the main aetiology for kataegis in prostate tumours

**Fig. 3:**
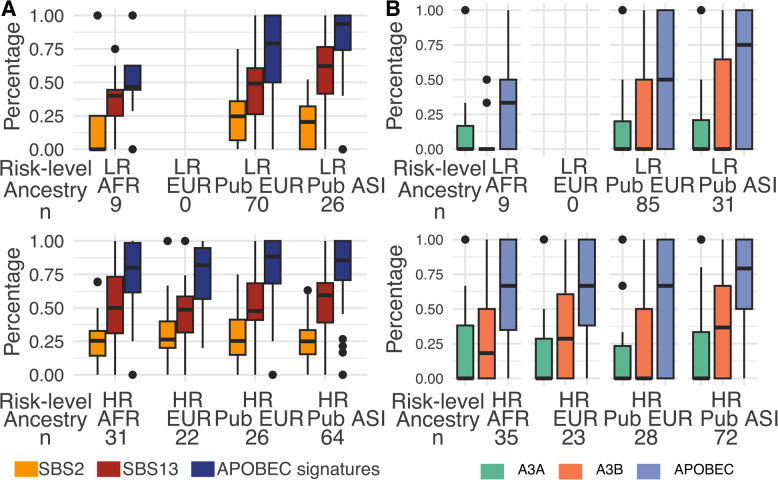
Attribution of kataegis to APOBEC enzyme activities.** A** Proportion of APOBEC-related single-base substitution (SBS) signatures per subgroup, including total APOBEC signatures (dark blue), SBS2 (gold) and SBS13 (red), with hyper-kataegic tumours excluded from the signature analysis. **B** Proportion of APOBEC-enriched kataegis per group, including APOBEC (blue-purple) determined by T*C*W motifs, and further the APOBEC3A (A3A, green) and APOBEC3B (A3B, orange) with YT*C*W and RT*C*W motifs, respectively. Patient ancestries are labelled as African (AFR), European (EUR), and Asian (ASI), with the prefix ‘Pub’ added for public data. Cancer risk levels are defined as low-risk (LR, ISUP GG1–2) and high-risk (HR, ISUP GG3–5) clinicopathological presentation. The number of patients per group is labelled underneath, excluding outliers

Our analysis of kataegis aetiology identified APOBEC as the primary contributing factor to kataegis across ancestries, except for the LR group of African patients. Particularly, SBS2 and SBS13 signatures accounted for approximately 80% (median: 79.1–93.6% for eight subgroups; Fig. 3A; all SBS proportions in Additional file1: Fig. S2), consistent with previous report (81.7%) [5]. Consistently, more than 50% (51.6–71.8%) of kataegis were APOBEC-enriched identified based on the motif preferences (Fig. 3B). Between ancestries, the LR group of Asian patients exhibited significantly more APOBEC-enriched kataegis than other ancestries (Fisher’s exact test, FDRs = 0.03 and 0.04 for LR group of African and public European data, respectively). Between risk-levels, the LR group of African patients showed significantly less APOBEC-enriched kataegis than the HR group (Fisher’s exact test, *P* = 0.04).

After observing APOBEC as the main contribution of kataegis events, we conducted a focused comparison between the two APOBEC-related signatures SBS2 and SBS13. The predominance of SBS13 (median, 40–62% for eight subgroups) over SBS2 was observed with significance in the HR group of African patients, and in both LR and HR groups of public European and Asian data (Wilcoxon’s rank sum test, FDR = $\:4.13\times\:{10}^{-10}$– $\:5.17\times\:{10}^{-3}$; Fig. 3A). Different from the other groups, the LR group of African patients showed the lowest proportion of APOBEC-related SBS2, significantly lower than the HR group (median, 0% vs. 25.4%; Wilcoxon’s rank sum test, *P* = 0.05).

We further attributed kataegis to the two APOBEC enzymes A3A and A3B. We observed higher proportions of A3B enrichment in all groups except for the LR group of African patients, with significance observed for larger public European LR and Asian LR/HR data (Wilcoxson’s rank sum test, FDRs = $\:7.04\times\:{10}^{-4}$–0.04; Fig. 3B). The predominant attribution to A3B was also observed in the early-onset PCa [48] but differs from the previous observation in hypermutated samples where A3A was strongly associated [42], probably because our samples exhibit lower levels of APOBEC activity. This argument is supported by the observation that APOBEC-related signatures were exclusively within kataegic SNVs and not from genome-wide SNVs (Additional file1: Fig. S3). Also, our PCa patients showed no *APOBEC3A* and *APOBEC3B* germline predispositions, as previously reported in the early-onset PCa [48] and other cancers [5, 49, 50], including rs12628403 [5], rs1014971 [50], and rs2142833 [5]. Kataegis status was not associated with somatic CNVs in *APOBEC3A* and *APOBEC3B* genes and regions within and between the genes. These findings align with the low frequency and burden of kataegis observed in PCa.

### Genomic rearrangement processes of ancestry predominant kataegis

**Fig. 4:**
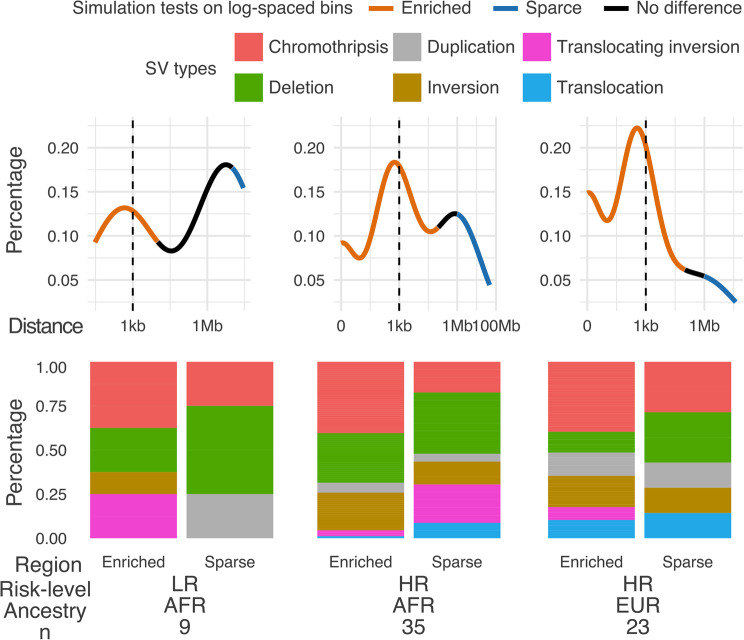
Distances between kataegis and proximal SVs. The top lines represent the density of SVs along the distance to proximal kataegis events. The bottom bar charts show proportion of SVs per type in kataegis enriched regions and sparse regions per patient group. Colours of the line show whether kataegis is significantly enriched (blue) or sparse (orange) within a region compared to simulations (simulation tests on log-spaced bins, FDR = $\:2.92\times\:{10}^{-3}$–0.01). Patient ancestries are labelled as African (AFR) and European (EUR). Cancer risk levels are defined as low-risk (LR, ISUP GG1–2) and high-risk (HR, ISUP GG3–5) clinicopathological presentation. The number of patients per group is labelled underneath excluding outliers

Having observed a close association between SV and kataegis abundance, we sought to further determine their distributions across the tumour genome. Kataegis events observed across ancestries and risk levels were significantly enriched around SV breakpoints, with 50% (413/831) within a 10 kb distance, 40% (335/831) within a 1-kb distance, and 13% spanning across SV breakpoints (109/831). Comparing kataegis to simulated kataegis events (1,000 times) with randomly selected non-clustering SNVs, we defined the ranges where kataegis were significantly enriched or sparse from an SV. Kataegis were significantly enriched around SV regions with varying ranges (0–10 kb to 0–1 Mb) and sparse at distances beyond 10 Mb or 100 Mb between groups of ancestries and risk levels (simulation tests on log-spaced bins, FDR = $\:2.92\times\:{10}^{-3}$–0.01; Fig. 4, Additional file1: Fig. S4-S6). We categorised kataegis to be SV-associated and independent for events located within enriched and sparse regions, respectively. The two types of kataegis varied in proportions between risk levels and between African and European ancestries. More SV-associated kataegis was observed in HR over LR groups (Fisher’s exact test, public European data, FDR = $\:4.01\times\:{10}^{-3}$), and in the HR groups of European over African patients (Fisher’s exact one-way test, *P* = 0.03). Focusing on SV types, chromothripsis was significantly enriched around kataegis (Fisher’s exact test, FDR = 0.04; Fig. 4), aligned with previous findings [6]. Conversely, more translocations were located distant to kataegis, with significance detected in the European public data (FDR = $\:1.17\times\:{10}^{-4}$).

**Fig. 5:**
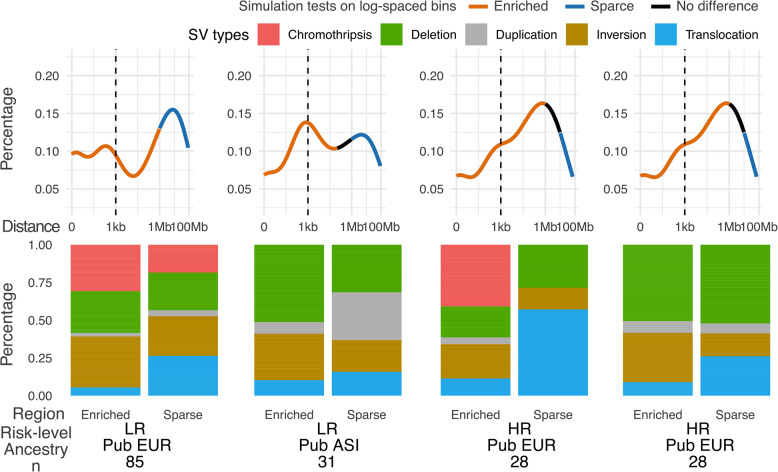
Proportion of structural variants (SV) signatures. Genome-wide SV signatures are identified from kataegis positive (+) and negative (-) prostate tumours of high-risk PCa (ISUP GG3–5) derived from Africans (AFR, *n* = 77) and Europeans (EUR, *n* = 48). Proportion of SV signatures per tumour (column) is defined as SV2 (blue), SV4 (orange), SV10 (green) and the others (grey). The number of patients per group is labelled underneath, excluding outliers

The analysis of genome-wide SV signatures for HR groups of the study cohort revealed an association between translocation SV type and kataegis. Compared to kataegis-negative tumours, kataegis-positives from both ancestries exhibited significantly lower proportion and less presence of the predominant SV2 signature and higher proportions and/or more presences of SV4 and SV10 (Fisher’s one-way exact test, FDR = $\:1.34\times\:{10}^{-4}$–0.01; Wilcoxon’s rank sum test, FDR = $\:9.84\:\times\:{10}^{-4}$– $\:8.68\:\times\:{10}^{-3}$; Fig. 5; all SV signatures identified in Additional file1: Fig. S7). According to the COSMIC SV signature database [41], simple translocations and clustered translocations are the primary components of SV2 and SV4, respectively, while SV10 encompass simple rearrangements of other types. These suggest kataegis-positive prostate tumours characterised by an increase in clustered translocations alongside non-clustered SVs of other types.

### Differential evolution of prostate tumour kataegis events between ancestries

**Fig. 6:**
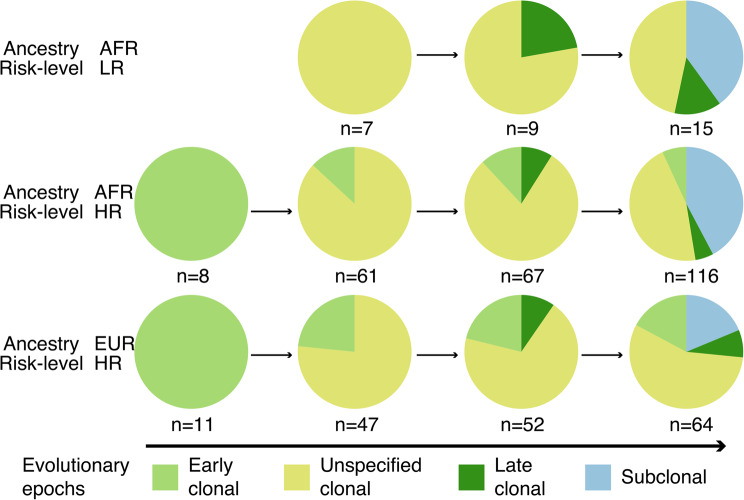
Evolution of kataegis events. Evolutionary kataegis were identified between cancer patients by ancestry, African (AFR) or European (EUR), and cancer risk, low-risk (LR, ISUP GG1–2) or high-risk (HR, ISUP GG3–5). The evolution of kataegis events is shown by their proportion along the development of cancer. The number of kataegis event is labelled underneath, excluding those identified in outliers

We revealed the uneven rise of kataegis across different evolutionary timeframes by assigning kataegis to clonal epochs (early, late, and unspecified) and the subclonal epoch for the study cohort (Additional file1: Supplementary methods; Additional file2: Table S7). Among the clonal SNVs originating from the clonal tumour cells, unspecified clonal SNVs were in diploid chromosomal regions. In regions where copy number was gained, early and late clonal SNVs were discernible, originating before and after the copy number gain, respectively. Both ancestries showed a bias towards clonal origins (65.0% clonal kataegis, 128/197; Fig. 6). The clonal proportion of kataegis was significantly higher than that of genome-wide SNVs (median, 100% vs. 68.3%; paired Wilcoxon’s rank sum test, *P* = 0.01), aligning with the clonal origin of chromothripsis [5] that could arise along with kataegis during telomere crisis [14]. This clonal bias of kataegis appears to be unreported in previous PCa studies [5, 15], while subclonal bias was reported for cancers with high kataegis burdens, excluding PCa [5]. Between ancestries, early clonal kataegis events were more frequent in European patients studied (EUR = 17.2% vs. AFR = 6.9%, Fisher’s exact test on HR groups, *P* = 0.04). In contrast, African-derived tumours exhibited an increased proportion of subclonal kataegis in both LR and HR groups; the latter showed significance when compared to the European patients (42% vs. 19%, Fisher’s exact test on HR groups, *P* = $\:1.67\:\times\:{10}^{-3}$). These findings suggest ancestral specific dynamics during carcinogenesis.

## Discussion

Kataegis is largely overlooked in PCa research due to its low frequency and burden compared to other cancer types, such as bladder and lung cancer [5]. To the best of our knowledge, no study has investigated the potential contribution of kataegis to ancestrally associated PCa health disparities. Here, using a unique multi-ancestral PCa resource, including southern African men representing the highest global region for PCa-associated mortality, complemented with published data [29–31], we present a detailed characterisation of kataegis features in prostate tumours, highlighting its implications in worse clinical outcomes and ancestrally different mutational processes. We observed prostate tumours exhibiting kataegis, often accompanied by cancer driver mutations and elevated genomic instability, are linked to adverse clinical outcomes. Tumours derived from African patients exhibited a higher proportion of kataegis independent of SVs and later occurrence in subclones. Among African patients, the proportion of kataegis attributed to APOBEC varied between cancer risks. These findings refine the earlier findings of ancestry-related cancer progression trajectories [17] by emphasising disparities in hypermutations, further underscoring the importance of African-inclusive investigations.

Furthermore, we propose kataegis as an indicator of adverse PCa which is independent of both ancestry and risk level. The similar prevalence of kataegis between risk levels highlights the limitation of current cancer grading which failed to detect any morphological or physical changes resulting from the interplay of kataegis, cancer drivers and genomic instability. Patients with kataegis-positive tumours may be recommended for more frequent monitoring during the remission period, as with a potentially higher metastatic risk. The heightened metastatic risk may be driven by genomic instability [51] and the two co-occurrent oncogenes of kataegis, *RBFOX1* and *TP53* [52, 53]. Also, PSA levels, known to be implicated in bone metastasis via the stimulation of osteoprotegerin [54], are found to rise in African patients with kataegis-positive aggressive PCa. However, our observation has challenged a previous statement that kataegis is a marker of good prognosis for BRCA [12]. The BRCA study showed significantly shorter survival time for patients with kataegis but proposed that aging might be the driving force. Therefore, further follow-up data from African patients is required for investigation, ideally in a large cohort to exclude potential confounding by age. Besides, we propose that the implication of kataegis in prostate tumours progression is different from early-onset PCa, BRCA and other cancer types with high kataegis burden, despite sharing features including elevated genomic instability, close association with SVs, and attribution to APOBEC enzyme activity [5, 11, 48]. Yet we observed no germline predisposition effects caused by *A3B* germline deletion [48, 49], partly because of its low frequency in African and European populations (0.9% and 6% respectively, vs. 22.5% worldwide) [55, 56]. Also, only the clustered mutations are attributed to APOBEC enzyme activity, which could be (i) the consequence of gradual dysregulation of APOBECs over the course of cancer progression [57], given most kataegis are clonal events; (ii) the technical difficulty in identification of low contribution of signatures [44].

Our African-inclusive study design has revealed ancestral disparities in kataegis development through evolutionary timing and mutational processes. Our evolutionary analyses have shown clonal kataegis predominated irrespective of patient ancestry. In particular, European ancestry has exhibited the high proportion of early clonal kataegis, indicating an implication in cancer initiation. In contrast, the subclonal kataegis identified in this study are notably biased towards African patients, regardless of clinicopathological presentation, suggesting a high level of genomic instability in cancer and, therefore, marked tumour heterogeneity and associated chemoresistance [58]. However, we acknowledge that our computational estimation of subclonal kataegis is a simplified model, further investigation with more sequencing techniques may help discern subclones and multiclonal origins, the prevalence of which is unknown to African patients.

**Fig. 7:**
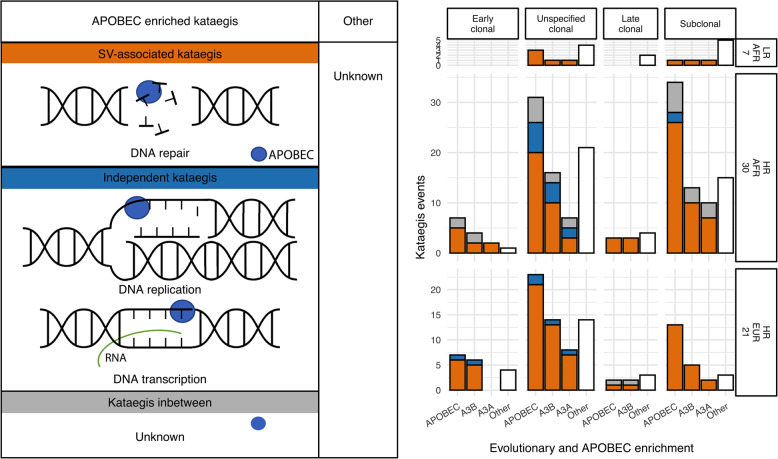
Ancestrally distinct kataegis development proposed in prostate cancer. For APOBEC enriched kataegis, SV-associated kataegis (orange) may arise during double-strand breaks (DSBs) repair, break induced replication (BIR) and telomere crisis, while independent kataegis (blue) may arise from dispersed APOBEC3 activity, which could happen at R-loop during transcription and lagging strand of DNA replication. Besides, kataegis in-between (grey) and non-APOBEC kataegis (white) remained unknown for mechanisms. We propose that these kateagis occur at different rates (indicated by bar plots) during the tumour evolution of African (AFR) vs. European (EUR) derived prostate tumours. Cancer risk levels are defined as low-risk (LR, ISUP GG1–2) and high-risk (HR, ISUP GG3–5) clinicopathological presentation. The number of patients per group is labelled underneath, excluding outliers

Additionally, we describe two kataegis mutational mechanisms as SV-associated and independent, observing varying proportions by ancestry with the former significantly more frequent in European than African patients. While kataegis are attributed to APOBEC deamination of cytosines from exposed ssDNA, mostly to APOBEC3B observed in this study, the deamination may take place under different processes for the two kataegis types (Fig. 7). We speculate that SV-associated kataegis, higher proportion in APOBEC-enriched kataegis (Fisher’s exact test, *P* = $\:9.65\times\:{10}^{-3}$), could have arisen during telomere crisis [14] and double-strand breaks (DSBs) repair mechanisms, such as break-induced replication (BIR) [59–61], as well as non-homologous end-joining (NHEJ) and alternative end-joining (A-EJ) concerning to the close association with chromothripsis [62]. Several lines of evidence indicate that chromothripsis-associated kataegis is induced by tumour crisis. Firstly, A3B, the major cause of the observed kataegis, is responsible for the cytosine deamination during chromothripsis breakpoints [13]. Secondly, the concurrence of driver mutations in *TP53*, a cell-cycle checkpoint gene, observed in European patients from this study supports the hypothesis that telomere crisis may result in chromothripsis-associated kataegis bypassing a cell cycle checkpoint due to checkpoint deficiency [14]. Also, significantly shorter telomere length has been observed in kataegis-positive tumours derived from the low-risk group of African patients and has been previously reported for the aggressive tumours derived from African men [63]. Conversely, we hypothesise that SV-independent kataegis, which we found to be more common in African ancestrally derived tumours, may arise on R loops in transcription bubbles or on the lagging strand of the DNA replication fork [64, 65]. The transcription and replication may be interplayed as R-loops in one of the sources that increase replication stress, leading to an elevated exposure of ssDNA at the replication fork [66]. We acknowledge, however, that our proposed hypotheses require further validation of cell experiments, such as DNA/RNA immunoprecipitation sequencing (DRIP)–R-loop experiments. Besides the two types, APOBEC kataegis in-between were more observed in African patients, suggesting unknown aetiologies requesting further studies. Altogether, these findings suggest divergent tumour pathways to some extent between ancestries.

While this study provides novel insights into kataegis in relation to ancestries and cancer aggressiveness, several limitations must be acknowledged. The lack of relevant data has hindered further validation or investigation, although this has been mitigated by integrating public cohorts. The clinical implications of kataegis for African patients need future research due to a lack of African follow-up and validation data. More LR data derived from African patients are also required for differentiating features between cancer aggressiveness. To scrutinise the ancestrally shared and distinctive features of kataegis, we integrated publicly available PCa data from European and Asian ancestral patients. However, this study and public cohorts differ in their composition of cancer aggressiveness and variant identification pipelines. While our study cohort is biased towards very HR disease (ISUP GG4/5), the public European dataset focused on intermediate risk disease (82%, ISUP GG2/3) [30], and the public Asian data lacks ISUP GG 5 (0.5%, 1/207) [31]. Additionally, although we applied consistent methods for kataegis identification and downstream analyses, our somatic variant identification is more stringent due to filtering by a panel of normal samples. These limitations highlight not only areas for future research, but importantly underscores the need for tailored data collection and analysis.

## Conclusions

The available PCa whole genome cohort remains one of the largest of its kind for the African continent and benefits from the inclusion of clinically, technically, and analytically matched non-African data, allowing for direct, unbiased comparative analyses. Using this African inclusive resource, supported by published non-African data, enabled us to discern both universal (or shared) and ancestrally unique kataegis positive prostate tumour features, particularly with regards to advanced disease. Demonstrating heightened African-specific kataegis-associated heterogeneity, our study emphasises the need for further African inclusion, specifically to elucidate the potential of kataegis and APOBEC3 enzymes as biomarkers of targeted cancer therapy. Collectively, by elucidating the occurrence of kataegis from tumorigenesis to later subclonal stage in African and European patients, we highlight the significance of different underlying mutational processes between ancestries, which provides a valuable resource for targeted therapeutic interventions and emphasises the need for continued exploration of biological behaviours and environmental exposures in African patients.

## Supplementary Information

Supplementary Material 1.

Supplementary Material 2.

## Data Availability

Sequence data of the study cohort was made available via Data Access Committee (DAC) approval in accordance with project-specific access policies as outlined through the European Genome‐Phenome Archive (EGA; (https://www.ega-archive.org)) under overarching accession EGAS00001006425 (https://www.ega-archive.org/studies/EGAS00001006425), for the Southern African Prostate Cancer Study (SAPCS) Dataset EGAD00001009067 (https://www.ega-archive.org/datasets/EGAD00001009067) and Garvan/St Vincent’s Dataset EGAD00001009066 (https://www.ega-archive.org/datasets/EGAD00001009066) [67]. The analysed variant data of two public cohorts are available from the ICGC Data Portal (http://dcc.icgc.org/) for the European cohort [68] and from the Genome Sequence Archive for Human (http://bigd.big.ac.cn/gsa-human/) under accession number PRJCA001124 for the Asian cohort [69].

## Abbreviations

A-EJ: Alternative end-joining
A3A: APOBEC3A
A3B: APOBEC3B
AFR: African ancestry
ASI: Asian ancestry
BCR: Biochemical relapse
BIR: Break-induced replication
BRCA: Breast cancer
CN: Copy number
CNVs: Copy number variants
COSMIC: Catalogue of somatic mutations in cancer
CPGEA: Chinese prostate cancer genome and epigenome atlas
DRIP: DNA/RNA immunoprecipitation sequencing
DSBs: Double-strand breaks
EGA: European genome-phenome archive
EUR: European ancestry
FDR: False discovery rate
GG: Grade group
HR: High-risk, Grade group 3–5
ICGC: International cancer genome consortium
ISUP: International society of urological pathology
LR: Low-risk, Grade Group 1/2
MRI: Magnetic resonance imaging
NHEJ: Non-homologous end-joining
NMF: Nonnegative matrix factorisation
PCa: Prostate cancer
PCAWG: Pan-cancer analysis of whole genomes
PCF: Piecewise constant fitting
PGA: Percentage of genome alteration
PSA: Prostate specific antigen
SAPCS: Southern african prostate cancer study
SBS: Single base substitution
SNVs: Single nucleotide variants
ssDNAs: Single-strand DNAs
SV: Structural variants
TMB: Tumour mutational burden

## References

[1] Bray F, et al. Global cancer statistics 2022: GLOBOCAN estimates of incidence and mortality worldwide for 36 cancers in 185 countries. CA Cancer J Clin. 2024;74(3):229–63. 38572751 10.3322/caac.21834

[2] Lee KM, et al. Association between prediagnostic prostate-specific antigen and prostate cancer probability in Black and non-Hispanic White men. Cancer. 2024;130(2):224–31. 37927109 10.1002/cncr.34979

[3] Nair SS, et al. Why do African-American men face higher risks for lethal prostate cancer? Curr Opin Urol. 2022;32(1):96–101. 34798639 10.1097/MOU.0000000000000951 PMC8635247

[4] Nik-Zainal S, et al. The life history of 21 breast cancers. Cell. 2012;149(5):994–1007. 22608083 10.1016/j.cell.2012.04.023 PMC3428864

[5] Aaltonen LA, et al. Pan-cancer analysis of whole genomes. Nature. 2020;578(7793):82–93. 32025007 10.1038/s41586-020-1969-6 PMC7025898

[6] Taylor BJ, et al. DNA deaminases induce break-associated mutation showers with implication of APOBEC3B and 3A in breast cancer kataegis. elife. 2013;2:e00534. 23599896 10.7554/eLife.00534 PMC3628087

[7] Veerla S, Staaf J. Kataegis in clinical and molecular subgroups of primary breast cancer. npj Breast Cancer. 2024;10(1):32. 38658600 10.1038/s41523-024-00640-8 PMC11043427

[8] Chen L, et al. Deep whole-genome analysis of 494 hepatocellular carcinomas. Nature. 2024;627(8004):586–93. 38355797 10.1038/s41586-024-07054-3

[9] Alexandrov LB, et al. Signatures of mutational processes in human cancer. Nature. 2013;500(7463):415–21. 23945592 10.1038/nature12477 PMC3776390

[10] Ansari-Pour N, et al. Whole-genome analysis of Nigerian patients with breast cancer reveals ethnic-driven somatic evolution and distinct genomic subtypes. Nat Commun. 2021;12(1):6946. 34836952 10.1038/s41467-021-27079-w PMC8626467

[11] Jakobsdottir GM, et al. APOBEC3 mutational signatures are associated with extensive and diverse genomic instability across multiple tumour types. BMC Biol. 2022;20(1):117. 35597990 10.1186/s12915-022-01316-0 PMC9124393

[12] D’Antonio M, et al. Kataegis expression signature in breast cancer is associated with late onset, better prognosis, and higher HER2 levels. Cell Rep. 2016;16(3):672–83. 27373164 10.1016/j.celrep.2016.06.026 PMC4972030

[13] Maciejowski J, et al. APOBEC3-dependent kataegis and TREX1-driven chromothripsis during telomere crisis. Nat Genet. 2020;52(9):884–90. 32719516 10.1038/s41588-020-0667-5 PMC7484228

[14] Maciejowski J, et al. Chromothripsis and kataegis induced by telomere crisis. Cell. 2015;163(7):1641–54. 26687355 10.1016/j.cell.2015.11.054 PMC4687025

[15] Cooper CS, et al. Analysis of the genetic phylogeny of multifocal prostate cancer identifies multiple independent clonal expansions in neoplastic and morphologically normal prostate tissue. Nat Genet. 2015;47(4):367–72. 25730763 10.1038/ng.3221 PMC4380509

[16] Raj A, Stephens M, Pritchard JK. fastSTRUCTURE: variational inference of population structure in large SNP data sets. Genetics. 2014;197(2):573–89. 24700103 10.1534/genetics.114.164350 PMC4063916

[17] Jaratlerdsiri W, et al. African-specific molecular taxonomy of prostate cancer. Nature. 2022;609:552–9. 36045292 10.1038/s41586-022-05154-6 PMC9477733

[18] Patrick SM et al. Prostate cancer clinicopathological presentation in South-East Africa during the 2010 decade. JNCI: J Natl Cancer Inst, 2025: p. djaf117. 10.1093/jnci/djaf117 PMC12682384 40342095

[19] Jiang J, et al. Scaling for African Inclusion in High-Throughput Whole Cancer Genome Bioinformatic Workflows. Cancers. 2025;17(15):2481. 40805180 10.3390/cancers17152481 PMC12346427

[20] Li H, Durbin R. Fast and accurate short read alignment with Burrows–Wheeler transform. Bioinformatics. 2009;25(14):1754–60. 19451168 10.1093/bioinformatics/btp324 PMC2705234

[21] Chen S, et al. fastp: an ultra-fast all-in-one FASTQ preprocessor. Bioinformatics. 2018;34(17):i884–90. 30423086 10.1093/bioinformatics/bty560 PMC6129281

[22] Faust GG, Hall IM. SAMBLASTER: fast duplicate marking and structural variant read extraction. Bioinformatics. 2014;30(17):2503–5. 24812344 10.1093/bioinformatics/btu314 PMC4147885

[23] DePristo MA, et al. A framework for variation discovery and genotyping using next-generation DNA sequencing data. Nat Genet. 2011;43(5):491–8. 21478889 10.1038/ng.806 PMC3083463

[24] Cameron DL, et al. GRIDSS2: comprehensive characterisation of somatic structural variation using single breakend variants and structural variant phasing. Genome Biol. 2021;22(1):202. 34253237 10.1186/s13059-021-02423-x PMC8274009

[25] Chen X, et al. Manta: rapid detection of structural variants and indels for germline and cancer sequencing applications. Bioinformatics. 2016;32(8):1220–2. 26647377 10.1093/bioinformatics/btv710

[26] Deshwar AG, et al. PhyloWGS: Reconstructing subclonal composition and evolution from whole-genome sequencing of tumors. Genome Biol. 2015;16(1):35. 25786235 10.1186/s13059-015-0602-8 PMC4359439

[27] Ha G, et al. TITAN: inference of copy number architectures in clonal cell populations from tumor whole-genome sequence data. Genome Res. 2014;24(11):1881–93. 25060187 10.1101/gr.180281.114 PMC4216928

[28] Gerstung M, et al. The evolutionary history of 2,658 cancers. Nature. 2020;578(7793):122–8. 32025013 10.1038/s41586-019-1907-7 PMC7054212

[29] Zhang J, et al. The international cancer genome consortium data portal. Nat Biotechnol. 2019;37(4):367–9. 30877282 10.1038/s41587-019-0055-9

[30] Fraser M, et al. Genomic hallmarks of localized, non-indolent prostate cancer. Nature. 2017;541(7637):359–64. 28068672 10.1038/nature20788

[31] Li J, et al. A genomic and epigenomic atlas of prostate cancer in Asian populations. Nature. 2020;580(7801):93–9. 32238934 10.1038/s41586-020-2135-x

[32] Lin X, et al. kataegis: an R package for identification and visualization of the genomic localized hypermutation regions using high-throughput sequencing. BMC Genomics. 2021;22(1):440. 34118871 10.1186/s12864-021-07696-x PMC8196519

[33] Team RC. R: A language and environment for statistical computing. R Foundation for Statistical Computing. No Title); 2013.

[34] Kassambara A. *ggpubr:‘ggplot2’based publication ready plots.* R package version, 2018: p. 2.

[35] Armenia J, et al. The long tail of oncogenic drivers in prostate cancer. Nat Genet. 2018;50(5):645–51. 29610475 10.1038/s41588-018-0078-z PMC6107367

[36] Ding L et al. *The roles of cyclin-dependent kinases in cell-cycle progression and therapeutic strategies in human breast cancer.* International journal of molecular sciences, 2020. 21(6): p. 1960. 10.3390/ijms21061960 PMC7139603 32183020

[37] Therneau TM, Lumley T. Package ‘survival’. R Top Doc. 2015;128(10):28–33.

[38] Kassambara A, et al. survminer: Drawing Survival Curves using ‘ggplot2’. R package version 0 4. 2021;9:p2021.

[39] Islam SA et al. Uncovering novel mutational signatures by de novo extraction with SigProfilerExtractor. Cell genomics, 2022. 2(11). 10.1016/j.xgen.2022.100179 PMC9646490 36388765

[40] Kuhn RM, Haussler D, Kent WJ. The UCSC genome browser and associated tools. Brief Bioinform. 2013;14(2):144–61. 22908213 10.1093/bib/bbs038 PMC3603215

[41] Everall A et al. *Comprehensive repertoire of the chromosomal alteration and mutational signatures across 16 cancer types from 10,983 cancer patients.* medRxiv, 2023: p. 2023.06. 07.23290970. 10.1038/s41588-025-02474-x PMC12987726 41688639

[42] Chan K, et al. An APOBEC3A hypermutation signature is distinguishable from the signature of background mutagenesis by APOBEC3B in human cancers. Nat Genet. 2015;47(9):1067–72. 26258849 10.1038/ng.3378 PMC4594173

[43] Roberts SA, et al. An APOBEC cytidine deaminase mutagenesis pattern is widespread in human cancers. Nat Genet. 2013;45(9):970–6. 23852170 10.1038/ng.2702 PMC3789062

[44] Zhang T, et al. APOBEC affects tumor evolution and age at onset of lung cancer in smokers. Nat Commun. 2025;16(1):4711. 40394004 10.1038/s41467-025-59923-8 PMC12092836

[45] Law EK et al. APOBEC3A catalyzes mutation and drives carcinogenesis in vivo. J Exp Med, 2020. 217(12). 10.1084/jem.20200261 PMC7953736 32870257

[46] Merriel SWD, et al. Systematic review and meta-analysis of the diagnostic accuracy of prostate-specific antigen (PSA) for the detection of prostate cancer in symptomatic patients. BMC Med. 2022;20(1):54. 35125113 10.1186/s12916-021-02230-y PMC8819971

[47] Milonas D, et al. The significance of prostate specific antigen persistence in prostate cancer risk groups on long-term oncological outcomes. Cancers. 2021;13(10):2453. 34070052 10.3390/cancers13102453 PMC8158093

[48] Gerhauser C, et al. Molecular evolution of early-onset prostate cancer identifies molecular risk markers and clinical trajectories. Cancer Cell. 2018;34(6):996–1011. e8. 30537516 10.1016/j.ccell.2018.10.016 PMC7444093

[49] Nik-Zainal S, et al. Association of a germline copy number polymorphism of APOBEC3A and APOBEC3B with burden of putative APOBEC-dependent mutations in breast cancer. Nat Genet. 2014;46(5):487–91. 24728294 10.1038/ng.2955 PMC4137149

[50] Middlebrooks CD, et al. Association of germline variants in the APOBEC3 region with cancer risk and enrichment with APOBEC-signature mutations in tumors. Nat Genet. 2016;48(11):1330–8. 27643540 10.1038/ng.3670 PMC6583788

[51] Fares J, et al. Molecular principles of metastasis: a hallmark of cancer revisited. Signal Transduct Target Therapy. 2020;5(1):28. 10.1038/s41392-020-0134-x PMC7067809 32296047

[52] Perron G, et al. Pan-cancer analysis of mRNA stability for decoding tumour post-transcriptional programs. Commun Biology. 2022;5(1):851. 10.1038/s42003-022-03796-w PMC9392771 35987939

[53] De Laere B, et al. TP53 outperforms other androgen receptor biomarkers to predict abiraterone or enzalutamide outcome in metastatic castration-resistant prostate cancer. Clin Cancer Res. 2019;25(6):1766–73. 30209161 10.1158/1078-0432.CCR-18-1943 PMC6330086

[54] Wong SK, et al. Prostate cancer and bone metastases: the underlying mechanisms. Int J Mol Sci. 2019;20(10):2587. 31137764 10.3390/ijms20102587 PMC6567184

[55] Kidd JM, et al. Population stratification of a common APOBEC gene deletion polymorphism. PLoS Genet. 2007;3(4):e63. 17447845 10.1371/journal.pgen.0030063 PMC1853121

[56] Petljak M, Maciejowski J. Molecular origins of APOBEC-associated mutations in cancer. DNA Repair (Amst). 2020;94:102905. 32818816 10.1016/j.dnarep.2020.102905 PMC7494591

[57] Mertz TM, et al. APOBEC-induced mutagenesis in cancer. Annu Rev Genet. 2022;56:229–52. 36028227 10.1146/annurev-genet-072920-035840

[58] Ashrafizadeh M, et al. Molecular panorama of therapy resistance in prostate cancer: a pre-clinical and bioinformatics analysis for clinical translation. Cancer Metastasis Rev. 2024;43(1):229–60. 38374496 10.1007/s10555-024-10168-9

[59] Elango R, et al. Repair of base damage within break-induced replication intermediates promotes kataegis associated with chromosome rearrangements. Nucleic Acids Res. 2019;47(18):9666–84. 31392335 10.1093/nar/gkz651 PMC6765108

[60] Sakofsky CJ, et al. Break-induced replication is a source of mutation clusters underlying kataegis. Cell Rep. 2014;7(5):1640–8. 24882007 10.1016/j.celrep.2014.04.053 PMC4274036

[61] Green AM, Weitzman MD. The spectrum of APOBEC3 activity: From anti-viral agents to anti-cancer opportunities. DNA Repair. 2019;83:102700. 31563041 10.1016/j.dnarep.2019.102700 PMC6876854

[62] Gelot C, Magdalou I, Lopez BS. Replication stress in Mammalian cells and its consequences for mitosis. Genes. 2015;6(2):267–98. 26010955 10.3390/genes6020267 PMC4488665

[63] Huang R, et al. The impact of telomere length on prostate cancer aggressiveness, genomic instability and health disparities. Sci Rep. 2024;14(1):7706. 38565642 10.1038/s41598-024-57566-1 PMC10987561

[64] McCann JL, et al. APOBEC3B regulates R-loops and promotes transcription-associated mutagenesis in cancer. Nat Genet. 2023;55(10):1721–34. 37735199 10.1038/s41588-023-01504-w PMC10562255

[65] Seplyarskiy VB, et al. APOBEC-induced mutations in human cancers are strongly enriched on the lagging DNA strand during replication. Genome Res. 2016;26(2):174–82. 26755635 10.1101/gr.197046.115 PMC4728370

[66] Saxena S, Zou L. Hallmarks of DNA replication stress. Mol Cell. 2022;82(12):2298–314. 35714587 10.1016/j.molcel.2022.05.004 PMC9219557

[67] Hayes VM, [Jiang JW, Gong J, Patrick T, Willet SM, Chew C, Lyons T, Haynes RJ, Pasqualim AM, Louw G, Kench M, Campbell JG, Horvath R, Chan LG, Wedge EKF, Sadsad DC, Brum R, Mutambirwa IS, Stricker SBA, Bornman PD, MSR]. African-specific molecular taxonomy of prostate cancer. Datasets; 2022. 10.1038/s41586-022-05154-6. The European Genome-phenome Archive. 10.1038/s41586-022-05154-6 PMC9477733 36045292

[68] Boutros PC, [Sabelnykova FM, Yamaguchi VY, Heisler TN, Livingstone LE, Huang J, Shiah V, Yousif YJ, Lin F, Masella X, Fox AP, Xie NS, Prokopec M, Berlin SD, Lalonde A, Ahmed E, Trudel M, Luo D, Beck X, Meng TA, Zhang A, D’Costa J, Denroche A, Kong RE, Espiritu H, Chua SM, Wong ML, Chong A, Sam T, Johns M, Timms J, Buchner L, Orain NB, Picard M, Hovington V, Murison H, Kron A, Harding K, P’ng NJ, Houlahan C, Chu KE, Lo KC, Nguyen B, Li F, Sun CH, de Borja RX, Cooper R, Hopkins CI, Govind JF, Fung SK, Waggott C, Green D, Haider J, Chan-Seng-Yue S, Jung MA, Wang E, Bergeron Z, Dal Pra A, Lacombe A, Collins L, Sahinalp CC, Lupien C, Fleshner M, He NE, Fradet HH, Tetu Y, van der Kwast B, McPherson T. JD, Bristow RG], *Genomic hallmarks of localized, non-indolent prostate cancer*. Dataset. International Cancer Genome Consortium (ICGC) Data Portal 10.1038/nature20788 (2017).

[69] Sun Y, [Xu LJ, Lee C, Ren HJ, Zi S, Zhang X, Wang Z, Yu H, Yang Y, Gao C, Hou X, Wang J, Yang L, Yang B, Ye Q, Zhou H, Lu T, Wang X, Qu Y, Yang M, Zhang Q, Shah W, Pehrsson NM, Wang EC, Wang S, Jiang Z, Zhu J, Chen Y, Chen R, Zhu H, Lian F, Li B, Zhang X, Wang Y, Wang C, Xiao Y, Jiang G, Yang J, Liang Y, Hou C, Han J, Chen C, Jiang M, Zhang N, Wu D, Yang S, Wang J, Chen T, Cai Y, Yang J, Xu W, Wang J, Gao S, Wang X. T], *A genomic and epigenomic atlas of prostate cancer in Asian populations.* Datasets. Genome Sequence Archive for Human; 2020. 10.1038/s41586-020-2135-x.

