# Supplementary material for: Single base focal hypermutation cooccurs with structural variation as an early event in advanced prostate tumourigenesis with ancestry specific independence: a multi-ancestral observational study
Source: Genome Med. 2026 Apr 25;18:87. doi: 10.1186/s13073-026-01647-5 (PMC13281657; doi:10.1186/s13073-026-01647-5)
Supplement: Supplementary file 1 — Supplementary Material 1. [file 13073_2026_1647_MOESM1_ESM.pdf]

Single base focal hypermutation cooccurs with structural variation as an early event in advanced prostate tumourigenesis with ancestry specific independence: a multi-ancestral observational study

Jue Jiang, Avraam Tapinos, Ruotian Huang, M.S. Riana Bornman, Phillip D. Stricker, Shingai B. A. Mutambirwa, David C. Wedge, Weerachai Jaratlerdsiri, and Vanessa M. Hayes

Supplementary methods and Figures

Supplementary methods

Kataegis identification with evolution

Evolutionary kataegis identification combined the steps developed by the PCAWG consortium [1] with our evolutionary timing analysis. To accurately capture co-occurring kataegic single nucleotide variants (SNVs), candidate events of evolutionary kataegis were identified from subsets of SNVs of each evolutionary epoch. Kataegis events were identified from each SNVs subset with following steps: *i*) estimating the threshold based on the number of SNVs of the subset; *ii*) adjusting the inter-mutational distance of SNVs within the subset by the piecewise constant fitting (PCF) model with default model parameters; *iii*) identifying candidate kataegis that met the threshold; and *iv*) filtering candidates to comply with at least one of the two kataegis characteristics, consistency of SNV types and identical chromosomal homologs [2, 3]. Below is more detailed steps.

Evolution of kataegis events

A kataegis event is believed to be the consequence of a single mutational process, so we identified kataegis events consisting of SNVs occurring from the same estimated evolutionary timing. Kataegis identification in a SNV subset could improve the number of false positives, as compared to traditional kataegis identification that uses genome-wide SNVs and may overestimate the closeness between neighbouring SNVs which may arise from different epochs. For each patient, SNVs were classified as clonal SNVs, subclonal SNVs, and unknown SNVs with unknown epoch. Clonal SNVs were further divided into early clonal, unspecified clonal, and late clonal SNVs. We separated early clonal, late clonal, and subclonal SNVs into different subsets while unspecified clonal and unknown SNVs were included in multiple subsets. For this study, the early clonal subset consisted of early clonal SNVs, unspecified clonal SNVs, and unknown SNVs. The late clonal subset consisted of late clonal SNVs, unspecified clonal SNVs, and unknown SNVs. The subclonal subset contained subclonal SNVs and unknown SNVs. Evolutionary timing of kataegis was assigned according to the timing of kataegic SNVs (Supplementary methods: Table. 1). In particular, an early clonal kataegis was defined as one from early clonal subset and contained at least one early clonal SNV. The same rule applied to late clonal kataegis and subclonal kataegis. Unspecified clonal kataegis mainly contained unspecified clonal SNVs and unknown SNVs if any. Unknown kataegis events contained only SNVs unknown to any epoch.

Table 1. Kataegis events assigned to each evolutionary epoch based on SNV composition

	**SNV composition***				
Kataegis	Early clonal SNVs	Late clonal SNVs	Unspecified clonal SNVs	Subclonal SNVs	Unknown SNVs
Early clonal kataegis	Yes	No	Optional	No	Optional
Late clonal kataegis	No	Yes	Optional	No	Optional
Unspecified clonal kataegis	No	No	Yes	No	Optional
Subclonal kataegis	No	No	No	Yes	Optional
Unknown kataegis	No	No	No	No	Yes

*Each row represents a kataegis evolutionary type with required SNVs noted as 'Yes' and optional SNVs noted as 'Optional'. SNVs should not be included denoted as 'No'.

Candidate kataegis thresholds

Particular thresholds were set to define both kataegis and evolutionary kataegis. Previously kataegis event was defined as having at least six consecutive SNVs within inter-mutational distance less than one kb after PCF adjustment [3], without considering the overall mutational load. As kataegis described a focal hypermutation against scattered mutations in the background, here we followed the improved identification method illustrated by PCAWG consortium [1], using a mutational burden (mb) adjusted thresholds for candidate kataegis identification. Multi-nucleotides substitutions were also included, using the substitution of the first position. The threshold pair (*K, d*) was calculated using Equation 1. *K* is the minimum number of kataegic SNVs ranging from four to six, and *d* is inter-mutational distance with a maximum of one kb and narrower for high mutational burden.

Equation 1:

$$d \leq \frac{-ln(1 - \sqrt[K-1]{\frac{0.01}{mb}})}{\lambda}$$

Assuming that the inter-mutational distance (X) within a genome follows an exponential distribution with rate $\lambda = \frac{ln2}{median\ (X)}$, the probability of an inter-mutational distance less than or equal to d will be $p = P(X \leq d) = 1 - e^{-\lambda d}$. For a pair of neighbouring SNVs, we test whether their distance is less than *d* and regard it as a Bernoulli trial with the success probability denoted as p. For a kataegis event with K mutations, we expect a streak of $K - 1$ success in N (= mb) trails, with the probability $S(N, K-1) = N(1-p)p^{K-1}$ assuming $N >> K$ so that $N - K \approx N$. The equation does not consider the status of other inter-mutational distances being success or failure to meet the threshold. We limit the probability $S(N, K-1) \leq 0.01$ and assume $p << 1$.

We calculated the threshold for each patient from both the studied cohort and public cohorts for candidate kataegis. For the candidate of evolutionary kataegis, we calculated the threshold for each subset of SNVs of selected evolutionary epoch for each patient from the studied cohort. The threshold was calculated using Equation 1 with K values being 4, 5 or 6, and the threshold pair (K, d) was selected by first maximising d and then minimising K values while having a ceiling $d_{max} = 1\ kb$. As all tumours were of low to moderate TMB, all the thresholds were set as $K = 4$ and $d = 1\ kb$.

PCF model

Inter-mutational distance of SNVs was adjusted by PCF model for both kataegis and evolutionary kataegis identification. The PCF model was firstly introduced for copy number variant calling. The model smooths copy number variation with a local average value and uses a penalty parameter γ to limit the number of segments. In kataegis calling, the PCF model smoothed the change of inter-mutational distances of SNVs and set neighbouring SNVs with identical distance. This adjustment allows some tolerance of inter-mutational distances greater than 1 kb. To perform PCF adjustment, we used the core algorithms of *Kataegis* R package [4] inherited from the *copynumber* R package [5], with default parameters, $\gamma = 25$ and $k_{min} = 2$, which were trained using breast cancer genomes [2]. We checked the fitting of the parameters by comparing calling results through rainfall plots generated by *karyoploteR* package [6] in R.

Kataegis filtration

The identification of kataegis and evolutionary kataegis followed the filtration methods described by PCAWG consortium[1]. SNVs of a kataegis event need to meet at least one of the two following criteria. Criterion 1 is consistency of a substitution type and Criterion 2 is kataegic SNV phasing to be a single haplotype.

Criterion 1: consistency of a substitution type in a row

As kataegic SNVs of an event occur from a single mutational process, their mutated nucleotides are expected to be the same type, for example, cytosine. For each candidate, we first identified the longest consecutive nucleotide being mutated, with K denoted as the number of the SNVs of the longest type. We denoted p as the local context frequency of a particular nucleotide, and N as the total number of SNVs of the candidate event. The probability of the type of nucleotide being mutated K times in a row, represented as $S(N, K)$, was computed with Equation 2 using dynamic programming. For a candidate with two or more mutated types being the longest in a tie, we calculated the probabilities for all of them. The calculated probabilities of all the candidates were adjusted according to Benjamini and Hochberg, with the false discovery rate (q value) threshold at 0.1.

Equation 2:

$$S(N, K) = p^K + \sum_{j=1}^{K} p^{j-1}(1-p)S(N-j, K)$$

For example, we assume cytosine is the longest consecutive type being mutated in a kataegis candidate, which is the most common situation in kataegis. The replacement of a cytosine is regarded to be a success in as a Bernoulli trial with the probability p which equals to the local background frequency of cytosine; mutations on other nucleotides are regarded as a failure. For a candidate consisting of N kataegic SNVs, the probability of having K consecutive SNVs with cytosine being mutated is denoted as $S(N, K)$.

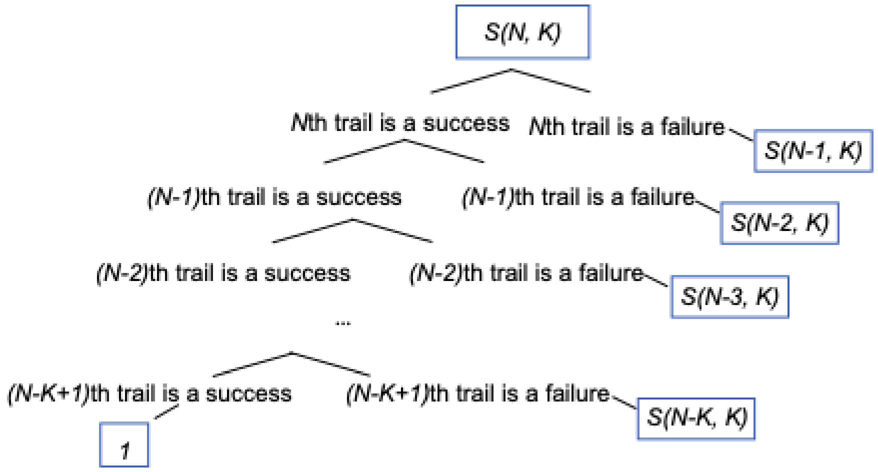

Fig. 1 All possible situations for $S(N, K)$

For computational efficiency, we used dynamic programming to calculate $S(N, K)$. The dynamic programming allows us to assume that the previous status like $S(N-1, K)$ is known and uses that status to calculate the current status $S(N, K)$ (Supplementary methods: Fig. 1). From the top, we first consider the Nth trail which can be either a success or a failure. In Situation 1, we assume the Nth trial is a failure. Given that, achieving consecutive K successes in N trials means having consecutive K successes in the first $N-1$ trail, whose probability is $S(N-1, K)$, and the overall probability of Situation 1 (including the Nth trial) is $(1-p)S(N-1, K)$. In Situation 2, we assume the Nth trail is a success but the $(N-1)$th trail is a failure. In Situation 2, having consecutive K successes in N trials is to have them in the first $(N-2)$ trail before testing the $(N-1)$th trail, whose probability is $S(N-2, K)$. The overall probability of Situation 2 is $p(1-p)S(N-2, K)$. By analogy, once a failure occurs in the last K trail to break the possibility of having consecutive K successes, reaching the consecutive K successes in N trials is to have these successes in previous trials. The total probability is the sum of probabilities from Situations 1 to K, which is $\sum_{j=1}^{K} p^{j-1}(1-p)S(N-j, K)$ where j can be 1, 2, …, K. On the other hand, if there is no failure in the last K trail, it means we have a streak of K successes at the last K trial; results of previous $(N-K)$ trails are inconsequential. The probability of this situation is p^K. By adding all the possibilities together, we obtain the equation of $S(N, K)$ as Equation 2.

Criterion 2: Single haplotype of kataegic SNVs

As APOBEC cytidine deaminases cause mutations on single strand DNA (ssDNA), we expect mutations are phased with each other. Generated by the GATK somatic short variant discovery

pipeline, physical phasing information included phasing genotypes and phasing group identity. We defined two mutations are in phase if *i*) they belong to the same phasing read group; *ii*) their phasing genotypes are the same; and *iii*) they are heterozygous.

References

1. Aaltonen, L.A., et al., *Pan-cancer analysis of whole genomes.* Nature, 2020. **578**(7793): p. 82-93.
2. Alexandrov, L.B., et al., *Signatures of mutational processes in human cancer.* Nature, 2013. **500**(7463): p. 415-421.
3. Nik-Zainal, S., et al., *The life history of 21 breast cancers.* Cell, 2012. **149**(5): p. 994-1007.
4. Lin, X., et al., *kataegis: an R package for identification and visualization of the genomic localized hypermutation regions using high-throughput sequencing.* BMC genomics, 2021. **22**(1): p. 440.
5. Nilsen, G., et al., *Copynumber: efficient algorithms for single-and multi-track copy number segmentation.* BMC genomics, 2012. **13**: p. 1-16.
6. Gel, B. and E. Serra, *karyoploteR: an R/Bioconductor package to plot customizable genomes displaying arbitrary data.* Bioinformatics, 2017. **33**(19): p. 3088-3090.

Supplementary Figures

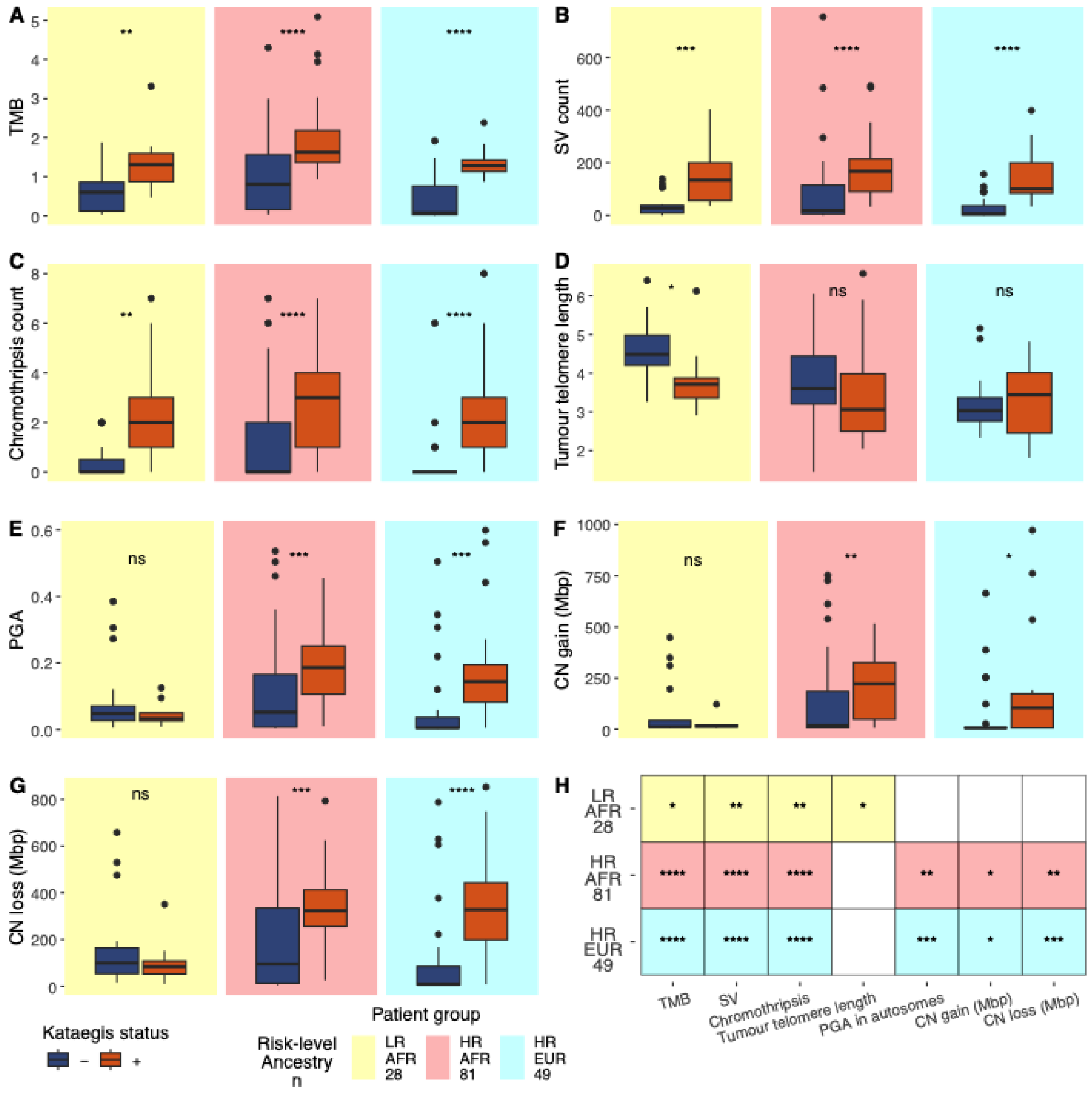

Fig. S1 Kataegis association with tumour genomic features in this multi-ethnic study cohort of 165 PCa patients distinguished by risk level and ancestry. The comparison of features between kataegis positive and negative tumours utilised Wilcoxon's rank sum test. A European derived hyper-kataegis outlier is excluded. Genomic features include: **A** Tumour Mutational Burden (TMB), **B** number of structural variants (SVs), **C** number of chromothripsis, **D** tumour telomere length, **E** percent genome alteration (PGA) with copy number (CN) variants in autosome, **F** CN gain and **G** loss in Mb. The comparison was made between kataegis positive (+) *vs* negative (-) by risk level defined as low-risk (LR, ISUP GG1-2) and high-risk (HR, ISUP GG3-5) clinicopathological presentation, and by ancestries including African (AFR) and European (EUR). Numbers underneath are the number of patients per group, excluding hyper-kataegis outliers. Significance is based on *P*-values (ns, not significant). **H** Significant levels of all genomic features after FDR adjustment of *P*-values depicted in A-G. Blank tiles indicate insignificance. Significant level descriptors include: *, *P*-value or FDR ≤ 0.05; **, ≤ 0.01; ***, ≤ 0.001; and ****, ≤ 0.0001.

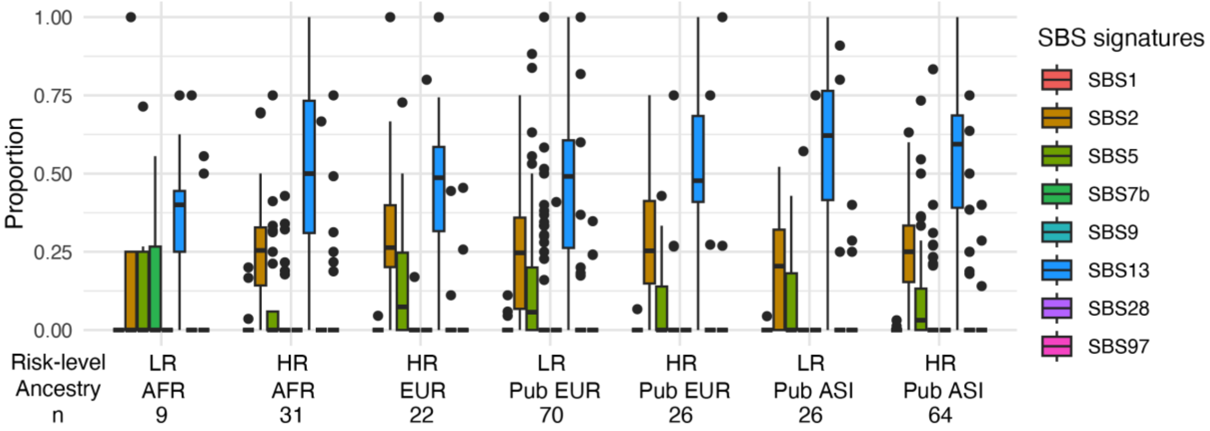

Fig. S2 Single-base substitution signatures decomposed from kataegic SNVs. Patient ancestries are labelled as African (AFR), European (EUR), and Asian (ASI), with the prefix 'Pub' added for public data. Cancer risk levels are defined as low-risk (LR) for ISUP GG1-2, and high-risk (HR) for ISUP GG3-5 clinicopathological presentation. Numbers underneath are the number of patients per group, excluding hyper-kataegis outliers.

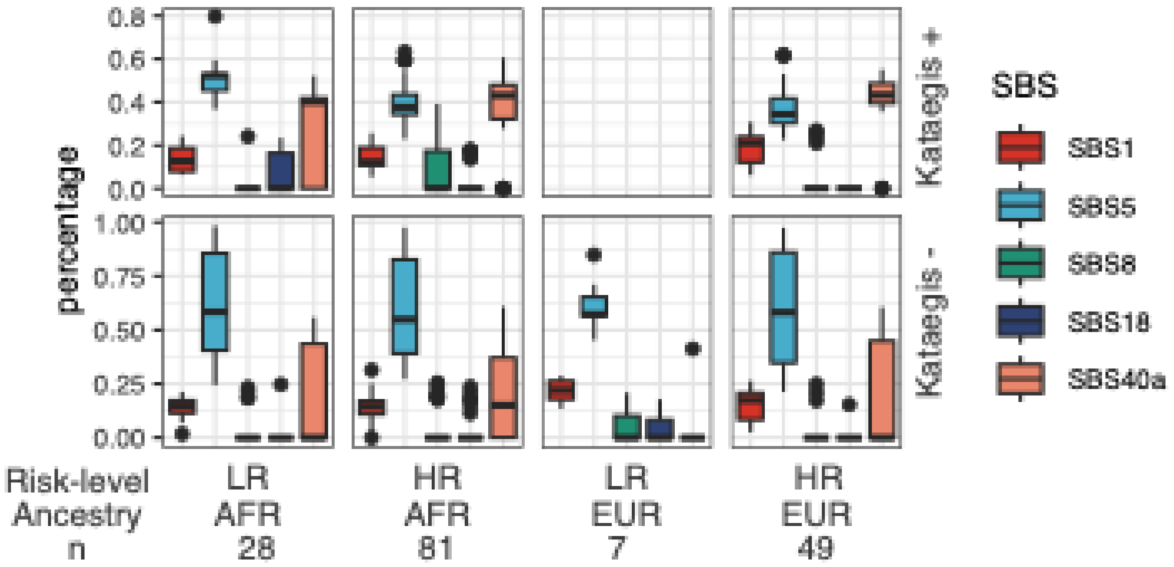

Fig. S3 Single-base substitution signatures decomposed from genome-wide SNVs of the study cohort. Patient ancestries are labelled as African (AFR), European (EUR). Cancer risk levels are defined as low-risk (LR) for ISUP GG1-2, and high-risk (HR) for ISUP GG3-5 clinicopathological presentation. Numbers underneath are the number of patients per group, excluding hyper-kataegis outliers.

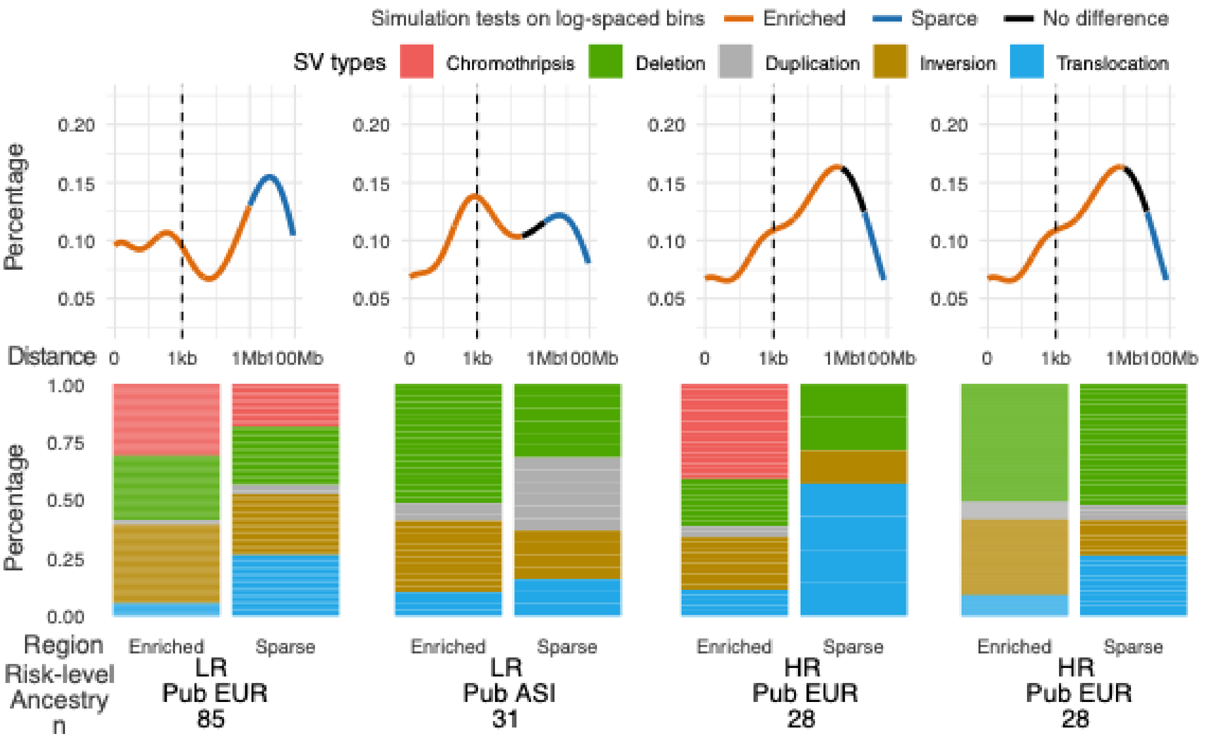

Fig. S4 Distances between kataegis and proximal SVs of public cohorts. The top lines represent density of SVs along the distance to a kataegis. Colours of the line show whether kataegis is significantly enriched or sparse to a region compared to simulated kataegis events. The bottom bars show proportion of SVs per type in kataegis enriched regions and sparse regions per patient group. Chromothripsis data are available for the public European patients, not for the public Asian patients. Patient ancestries are labelled as European (EUR) and Asian (ASI), with the prefix 'Pub' for public data. Cancer risk levels are defined as low-risk (LR, ISUP GG1-2) and high-risk (HR, ISUP GG3-5) clinicopathological presentation. The number of patients per group is labelled underneath excluding outliers.

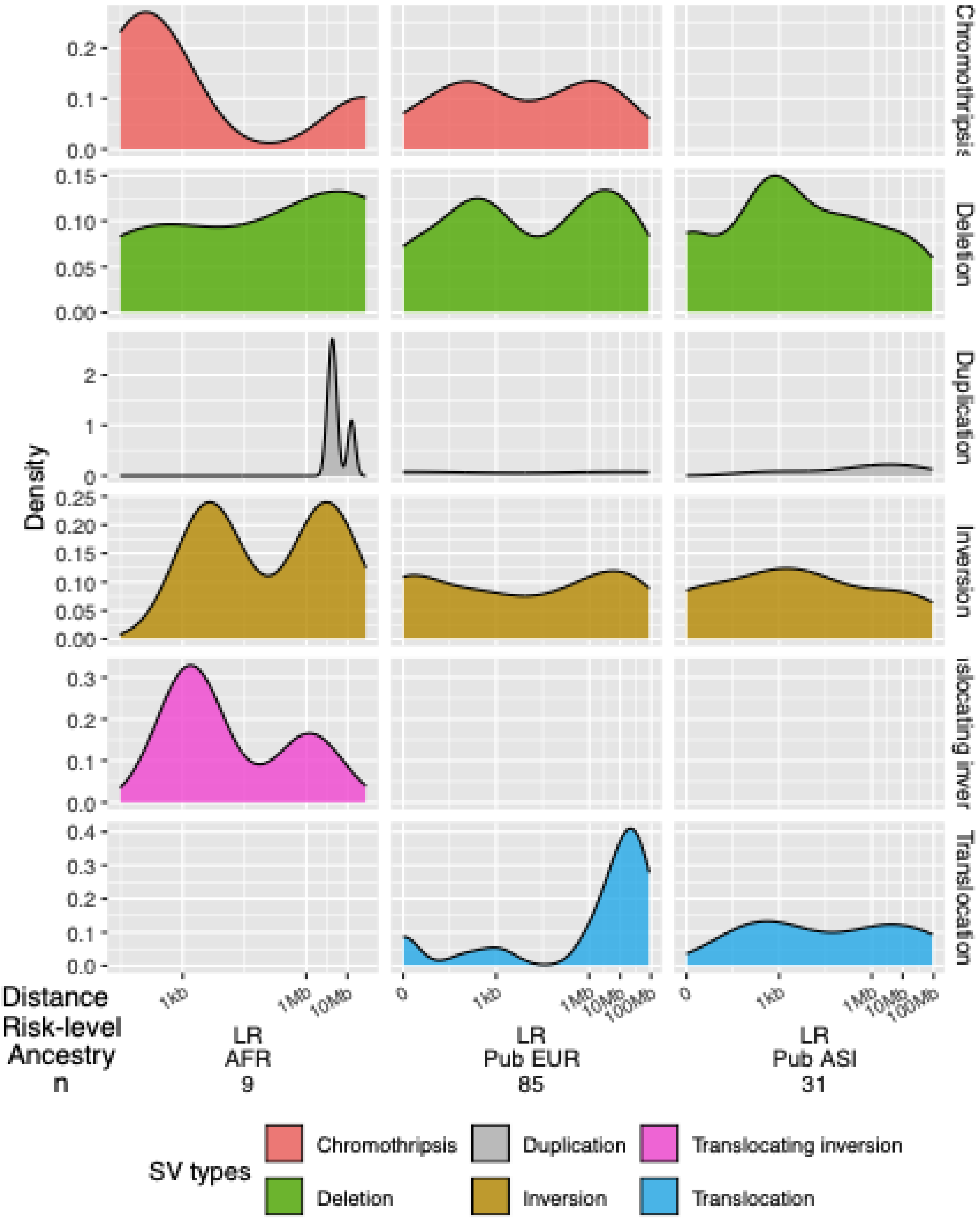

Fig. S5 Distribution of proximal SVs for each SV type to kataegis in low-risk (LR) groups**.** Patient ancestries are labelled as African (AFR), European (EUR), and Asian (ASI), with the prefix 'Pub' added for public data. Low-risk (LR) cancer risk level is defined as ISUP GG1-2 clinicopathological presentation. Numbers underneath are the number of patients per group excluding hyper-kataegis outliers.

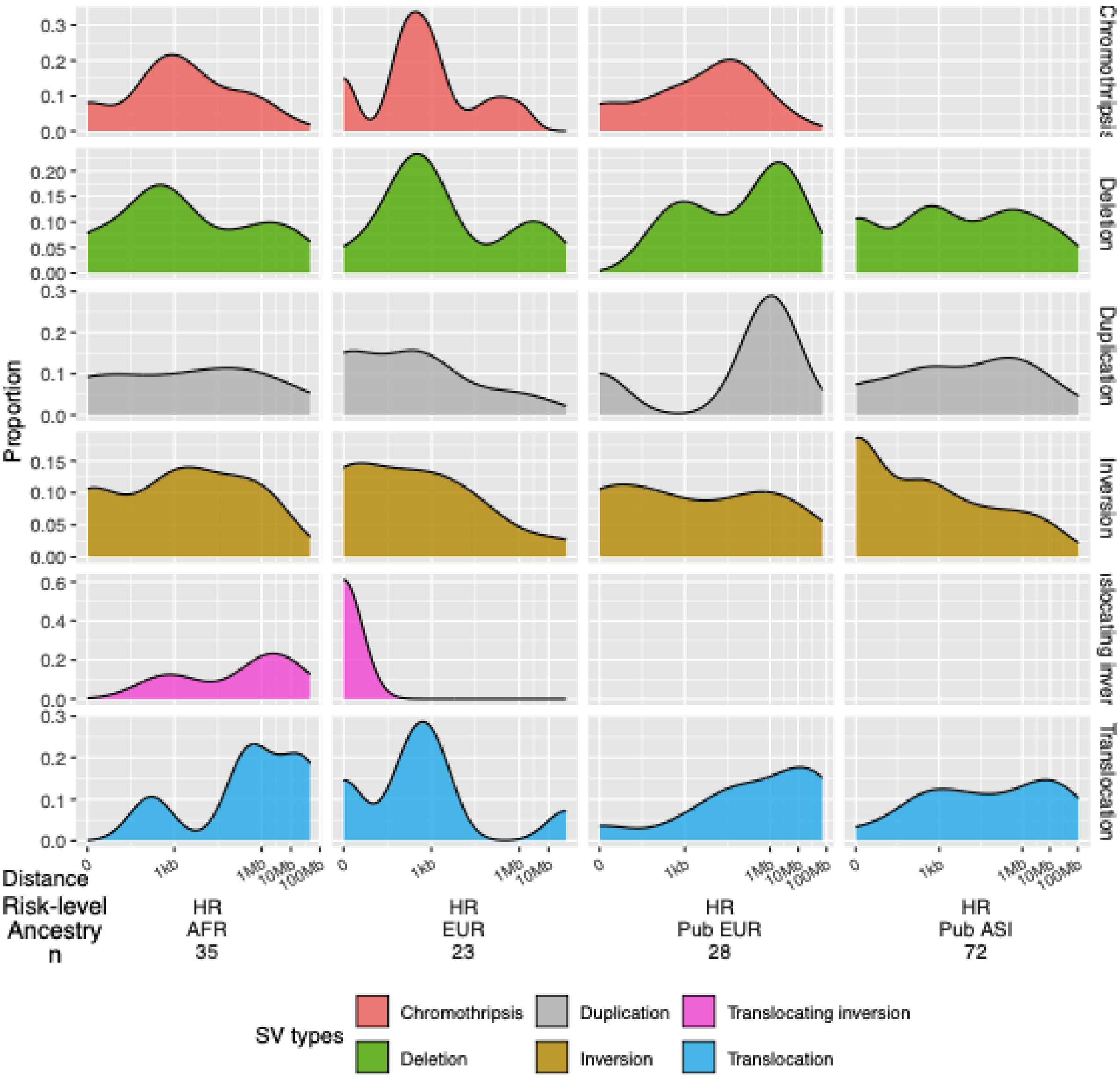

Fig. S6 Distribution of proximal SVs per SV type to kataegis in high-risk (HR) groups. Patient ancestries are labelled as African (AFR), European (EUR), and Asian (ASI), with the prefix 'Pub' added for public data. High-risk (HR) cancer risk level is defined as ISUP GG3-5 clinicopathological presentation. Numbers underneath are the number of patients per group excluding hyper-kataegis outliers.

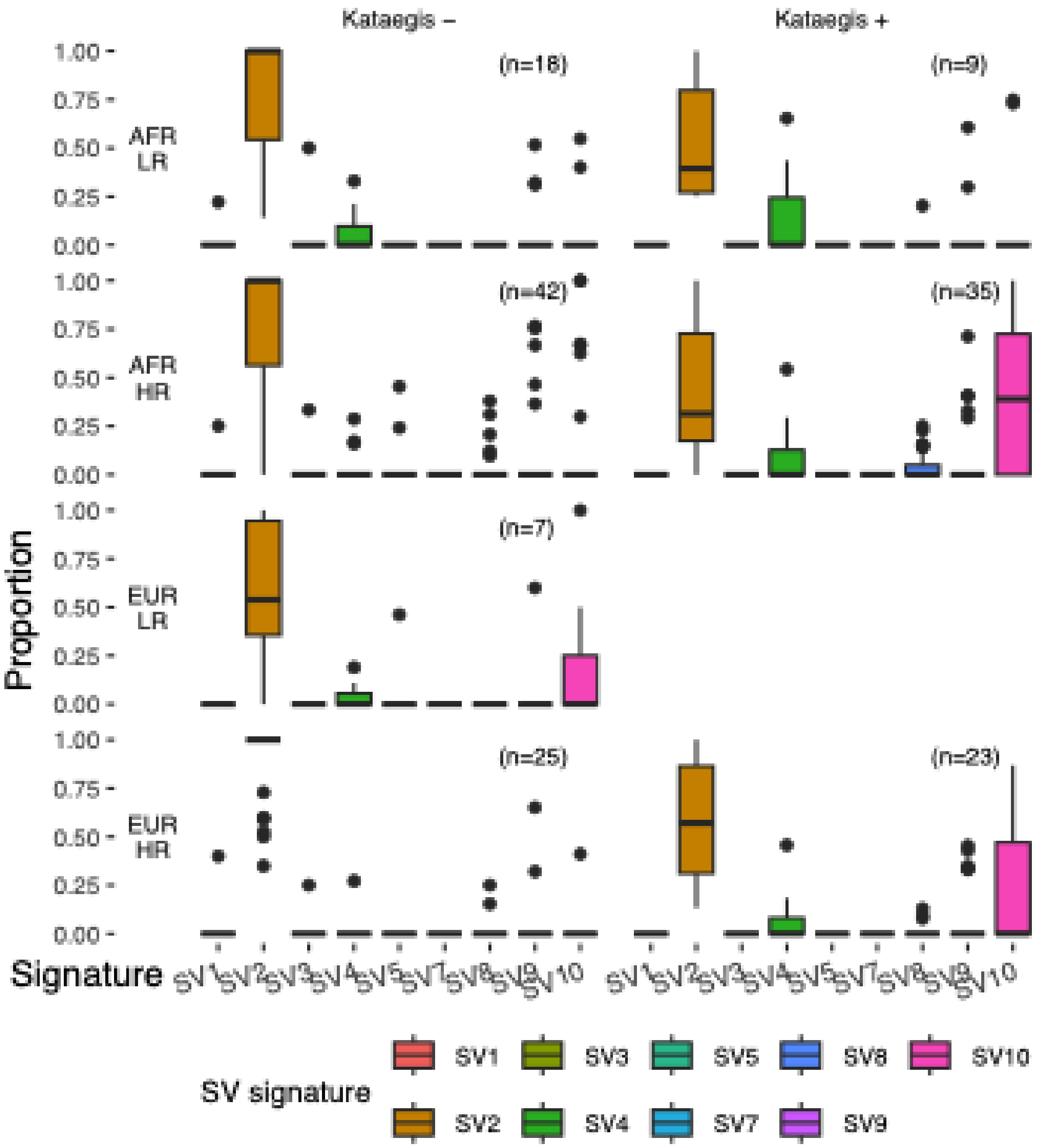

Fig. S7 Proportion of genome-wide SV signatures with respect to kataegis status. Patient ancestries are labelled as African (AFR), European (EUR), and Asian (ASI), with the prefix 'Pub' added for public data. Cancer risk levels are defined as low-risk (LR, ISUP GG1-2) and high-risk (HR, ISUP GG3-5) clinicopathological presentation. Hyper-kataegis outliers are excluded. The number of patients per group is labelled on the top right, excluding outliers.
